# A Review of Composite Phase Change Materials Based on Porous Silica Nanomaterials for Latent Heat Storage Applications

**DOI:** 10.3390/molecules26010241

**Published:** 2021-01-05

**Authors:** Raul-Augustin Mitran, Simona Ioniţǎ, Daniel Lincu, Daniela Berger, Cristian Matei

**Affiliations:** 1 “Ilie Murgulescu” Institute of Physical Chemistry, Romanian Academy, 202 Splaiul Indepedentei, 060021 Bucharest, Romania; ionitasimona05@gmail.com (S.I.); daniel.lincu1113a@gmail.com (D.L.); 2 Faculty of Applied Chemistry and Material Science, University “Politehnica” of Bucharest, 1-7 Polizu Street, 011061 Bucharest, Romania; danaberger01@yahoo.com (D.B.); cristi_matei@yahoo.com (C.M.)

**Keywords:** phase change materials, shape-stabilized, silica, porous, latent heat, thermal energy storage

## Abstract

Phase change materials (PCMs) can store thermal energy as latent heat through phase transitions. PCMs using the solid-liquid phase transition offer high 100–300 J g^−1^ enthalpy at constant temperature. However, pure compounds suffer from leakage, incongruent melting and crystallization, phase separation, and supercooling, which limit their heat storage capacity and reliability during multiple heating-cooling cycles. An appropriate approach to mitigating these drawbacks is the construction of composites as shape-stabilized phase change materials which retain their macroscopic solid shape even at temperatures above the melting point of the active heat storage compound. Shape-stabilized materials can be obtained by PCMs impregnation into porous matrices. Porous silica nanomaterials are promising matrices due to their high porosity and adsorption capacity, chemical and thermal stability and possibility of changing their structure through chemical synthesis. This review offers a first in-depth look at the various methods for obtaining composite PCMs using porous silica nanomaterials, their properties, and applications. The synthesis and properties of porous silica composites are presented based on the main classes of compounds which can act as heat storage materials (paraffins, fatty acids, polymers, small organic molecules, hydrated salts, molten salts and metals). The physico-chemical phenomena arising from the nanoconfinement of phase change materials into the silica pores are discussed from both theoretical and practical standpoints. The lessons learned so far in designing efficient composite PCMs using porous silica matrices are presented, as well as the future perspectives on improving the heat storage materials.

## 1. Introduction

The generation, transportation and usage of energy are intrinsically linked with human activities. Higher energy availability has led to increasing living standards and economic development, but it also negatively impacting the environment. Efficient energy generation, storage and use are therefore becoming increasingly important, since they play a large role in both energy and industrial applications [1,2]. Storing thermal energy in the form of heat or cold is an important segment of the energy infrastructure. Applications of thermal energy storage range from passively heated and cooled building, waste heat reutilization and renewable energy storage to creating better thermal insulation of transplant organs, food or electronics and battery heat management.

One of the most promising technologies for thermal energy storage is comprised of phase change materials (PCMs), which can reversibly store large amounts of heat and cold at constant operating temperature. The use of pure compounds as PCMs has a number of drawbacks, such as leakage during use, poor reliability during multiple operating cycles, corrosiveness, flammability, etc. Most of these undesired properties can be alleviated by creating composite materials consisting of a porous matrix and the active heat storage compound. Porous silica nanomaterials offer high porosity and adsorption capacity, chemical and thermal resistance, non-toxicity and their properties can be easily tailored towards specific applications through various synthetic pathways, making them ideal matrices for PCMs.

The field of porous silica matrices for phase change materials is a new and active area of research, with almost all reports published in the 2010–2020 decade. This work therefore aims to provide a comprehensive and up-to-date characterization of the materials and methods used to create nanocomposite phase change materials with porous silica matrices. The relevant properties for thermal energy storage and how they are influenced by the various factors involved in obtaining the nanocomposites are discussed, with a particular focus on the physico-chemical processes taking place under nanoconfinement. The effects of the type and properties of the porous silica matrices on the final thermal energy storage capacity are also addressed.

## 2. Thermal Energy Storage

Thermal energy storage (TES) is an important component in the area of sustainable energy production and in many industrial processes. While the use of naturally occurring materials to store thermal energy as heat or cold has taken place since prehistory, this is still an active research topic. There are three main strategies for TES: sensible, latent and chemical heat storage. Sensible heat storage denotes the energy lost or gained by heating or cooling a material respectively. The amount of heat lost or gained by a gram or a mole of material when changing its temperature with 1 °C represents its specific heat (c_p_). Liquid water has one of the highest specific heats, at 4.184 J g^−1^ K^−1^. However, water remains liquid in a short temperature range, so cheaper materials with high thermal stability ranges but low c_p_, such as concrete or rock, are also employed for sensible heat storage [3]. Latent heat storage relies on the energy exchange which takes place during a phase transition such as melting/crystallization or evaporation/condensation. Latent heat storage can accommodate large heat storage capacities at constant temperature. Materials used for latent storage are called phase change materials (PCM). Finally, chemical heat storage involves the reaction enthalpy to be used for heat storage. Since a practical TES system needs to be reversible, chemical heat storage often involves several reactions in a cycle, controlled by changes in temperature, pressure or reactant concentrations. Thus, a chemical storage system is often more complex than the alternatives. Needless to say, two or all three main strategies for heat storage can be combined in a practical system.

Each of the heat storing strategy has specific advantages and disadvantages. Sensible and latent storing are passive systems which do not require complex equipment or moving parts, thus reducing the cost of heat storage. However, sensible storage has low overall heat storage capacity. Specific heat capacity, the operating temperature range, cost, and density are thus the most important characteristics of a sensible heat storage material (Table 1). In contrast, latent heat storage offers higher energy densities at constant operating temperature. PCMs for latent storage can in theory use any phase transition between solid, liquid and gas phases. However, the change in volume accompanying the gas phase is very large, greatly reducing the volumetric storage capacity of such a system. In practice, only transitions between solid and liquid phases are employed. Solid-solid transitions have smaller change in molar volume, but also smaller transition enthalpy than solid-liquid transitions. The transition enthalpy and temperature are the main physical properties affecting the heat storage capacity. It should be noted that a direct correlation between melting points and the heat of fusion exists, with materials having higher melting points generally exhibiting higher enthalpies as well. Volume change on transition, cost, stability under repeated cycling, degree of supercooling, and vapor pressure also affect the thermal energy storage profiles of PCMs. Chemical heat storage is the most complex method for storing thermal energy. This method often involves one or more equilibrium chemical reactions with high enthalpy, in which one of the products is gaseous, being removed or added during heat charging and discharging [4]. The main advantages of chemical storage are its high specific energy storage capacity. In theory, the energy containing materials could be stored and transported at ambient temperature, without heat loss. However, most chemical storage reactions require elevated temperatures, thus doing so will decrease the effectiveness of the storage system.

A comparison between the three heat storage methods (Table 1) shows their strengths and weaknesses. The research interest in the area of thermal energy storage is justified by both latent and chemical storage, offering higher energy densities than sensible heat storage. The focus of this review is on latent heat storage using phase change materials, which can provide the low-cost, high thermal energy density when operating over a small temperature range.

### 2.1. Phase Change Materials

**Figure 1:**
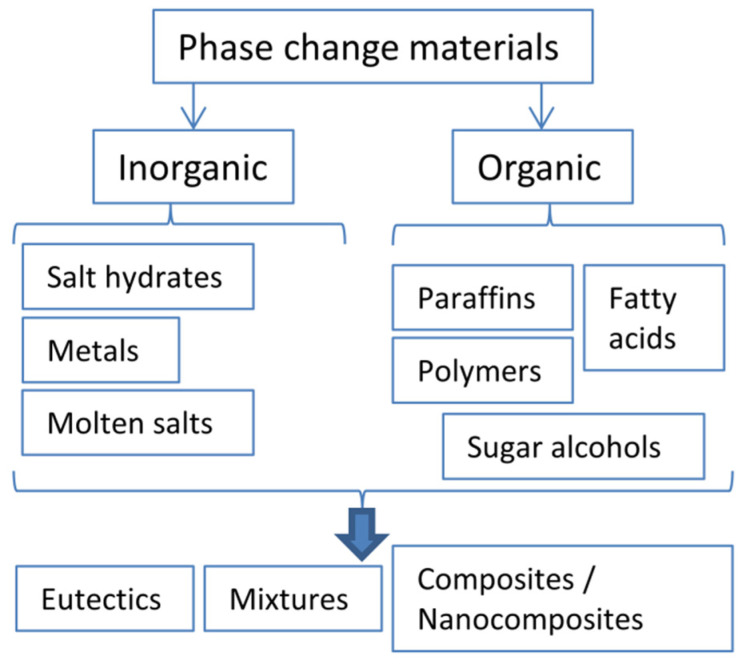
Classification of the main PCM classes.

Latent energy storage is carried out using phase change materials. A large number of substances can act as PCMs, provided they possess high heat of fusion values, a usable melting point, low supercooling and fast crystallization. PCMs should be thermally and chemically stable. The latent heat storage materials can be both organic and inorganic [5]. Organic PCMs can be further divided into paraffins, fatty acids, alcohols or amides, sugar alcohols and polymers such as poly ethylene glycol (Figure 1). The large heat of fusion for organic PCMs arises from either the attractive forces between the long *n*-alkyl chains or paraffins and fatty acid compounds or from strong intermolecular hydrogen bonding. Inorganic PCMs mainly consist of salt hydrates, molten salts, metals or other elementals, and compounds (oxides, hydroxides, carbonates, halides etc.). Prospective readers interested in a more detailed analysis of various PCMs are directed towards some comprehensive reviews on this topic [6,7,8,9,10,11]. It is also worth mentioning that PCMs can be used for other applications except heat storage, such as electronic and plasmonic devices [12,13,14].

Eutectic or non-eutectic mixtures of various PCM compounds are also used. Mixtures enable the creation of PCMs with desired melting points for each specific application. Some disadvantages remain however for most pure PCMs or their mixtures, including low thermal conductivity for most non-metals, leakage, and decreased capacity during use. A proposed solution to alleviating these drawbacks is the use of composites or nanocomposites. The incorporation of metals or carbon frameworks or nanoparticles can be used to increase thermal conductivity [15,16,17]. Shape-stabilized or form stable materials which retain their macroscopic solid shape even when the PCM is melted can be created. These shape-stabilized PCMs do not leak material during use, which greatly increases their lifetime by minimizing the loss of material, corrosiveness, and volume changes, which can also affect thermal contacts.

Two strategies for creating shape-stabilized PCMs have emerged. First, the PCM can be encapsulated with a shell of inert material [18,19,20,21]. Carbon materials or metal oxides are often employed as the shell material, while the PCM nanoparticles or microparticles make the core. The challenge is to get a uniform shell of the least thickness required to prevent PCM leakage. Increasing the radius of the particles decreases the amount of shell material, while simultaneously decreasing thermal transfer. As such, most examples of encapsulated phase change materials are in the micrometer range. The second approach consists of impregnating PCMs into high porosity matrices [22,23,24,25]. The liquid compounds are stabilized through capillary interactions. Impregnation allows for lower mass fractions of matrix material, leading to higher energy storage capacity. However, the phase change material can still be lost through evaporation. The most common porous matrices are based on carbon, polymers, silica or other oxides [26,27,28,29]. Porous silica nanomaterials offer high and tunable porosity, good thermal and chemical stability, non-toxicity, and abundant precursors. Thus, they will constitute the focus of this review.

### 2.2. Porous Silica Matrices

Impregnation of phase change materials into high porosity matrices is used to obtain shape-stabilized PCMs (ssPCMs). SsPCMs retain their macroscopic solid shape even when the active heat storage materials are melted. This is due to capillary forces between the heat storage phase and the matrix. Matrices with low pore sizes and high pore volumes increase the amount of encapsulated PCMs and therefore such composites have higher thermal energy storage capacity. Moreover, the capillary forces needed for shape-stabilization are smaller for lower pore diameters. Porous silica nanomaterials have attracted attention as ssPCMs matrices due to their high porosity, high thermal and chemical stability, and the ease with which their textural properties can be altered.

Porous silica materials can be divided into zeolites, mesoporous silica, silica nanoparticles, porous glass, aerogels, and xerogels. Zeolites are typically silicate materials which contain an ordered structure and micropores less than 2 nm in diameter [30,31]. While the size of pores can be precisely controlled, the small diameter makes then less suitable for PCM nanocomposite applications. Monodisperse silica nanoparticles can be obtained starting with diameters as low as 20 nm [32]. Even though the nanoparticles are not porous, their spherical and monodisperse nature gives rise to interparticle porosity. Porous matrices such as fumed silica can be obtained from the agglomeration of silica nanoparticles and used to obtain ssPCMs [33]. Silica aerogels and xerogels are one of the most porous materials ever obtained, with surface area up to 2000 m^2^ g^−1^ and densities as low as 0.003 g cm^−3^ [34,35]. Aerogels are also very good thermal insulators, having thermal conductivity values around 0.005 Wm^−1^ K^−1^ [36]. Aerogels and xerogels are obtained from a silica sol in which the solvent is replaced with a supercritical fluid or another solvent, respectively. Aerogels have a disordered and interconnected pore network left behind by the solvent in the initial silica gel material. Porous glass has a similar pore structure, although with less porosity than aerogels. Mesoporous silica nanomaterials (MSN) are one of the most versatile porous matrices [37]. MSN contain monodisperse mesopores in the 2–50 nm diameter range, which are usually ordered into hexagonal, lamellar or cubic arrangements [38]. MSN have thicker pore walls than aerogels and thus higher mechanical stability. The materials have 500–1000 m^2^ g^−1^ surface area and total mesopore volumes up to 3.5 cm^3^ g^−1^ [39].

**Figure 2:**
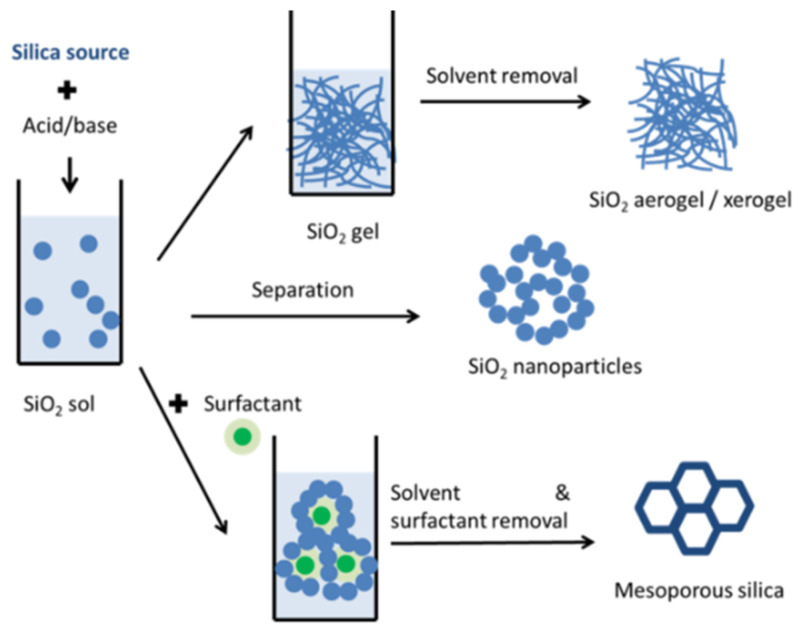
Schematic representation of porous silica solution synthesis methods.

The most common strategy for obtaining porous silica nanomaterials is sol-gel synthesis (Figure 2). A suitable silica precursor such as tetraethyl orthosilicate (TEOS) or sodium silicate is hydrolyzed in acid or basic media. The resulting silanol moieties condense and form silica [40,41]. The reaction media can be used to control the reaction in the case of nanoparticles, aerogels or xerogels. One or more solvent exchange steps are performed after the sol-gel reaction for aerogels and xerogels. The porosity of the resulting materials can be easily changed through the processing parameters (time, concentration, temperature, solvents, etc.) [42]. Mesoporous silica porosity is created by the addition of different surfactants and pore expanders during the sol generation or the gelling processes. The surfactants act as structure directing agents around which the silica framework is consolidated and they can be removed by calcination or solvent treatments. The use of surfactants as soft templates is different from their use to prevent nanoparticle agglomeration for other nanomaterials [43,44]. In this case, the pores are formed from the removal of the surfactants, while the classical silica nanoparticle synthesis does not involve the use of any surfactants [32].

Porous glass is commonly obtained using an acid soluble glass phase as a hard template. This soluble phase is removed by mineral acid treatment, leaving behind a disordered, porous structure [45]. Polymer nanoparticles can be used as a hard template, leading to the creation of silica hollow spheres [46,47].

The high surface area of the porous silica nanomaterials is covered with silanol moieties. These groups can participate in intermolecular H-bonding with suitable encapsulated molecules. A simple way to change the surface properties of the porous silica matrices is the introduction of functional organic groups through. This process is called functionalization, and it can be performed with different organosilane molecules either after the synthesis of the porous silica (post-functionalization) or during the porous silica synthesis (co-condensation) [48,49,50]. The functional organic groups can be used as starting points for creating complex organic molecules anchored to the silica surface [51,52]. Doping the silica framework with ad-atoms is another strategy used to control the properties of the porous matrices [53,54,55]. This method can also introduce electrostatic charges on the surface of the porous silica. For example, aluminum is widely used to create positively charge surfaces at neutral pH [56]. Introducing other compounds such as nanoparticles either in the silica framework or in a part of the pore volume can provide various new functions to the matrices. Examples include magnetic behavior [57,58], stimuli responsiveness [59,60], increased thermal stability, etc. [61,62].

Together with the ease of changing particle size and shape, pore size, arrangement and volume through modifying the synthesis parameters, these strategies make porous silica nanomaterials versatile matrices for PCMs.

### 2.3. Porous Silica Nanocomposites: Desired Properties and Synthesis

The use of any porous silica–based thermal energy system is predicated on its properties. The capacity for thermal energy storage and the transition temperature are the most important thermal properties for any phase change materials. High specific heat and thermal conductivity are also desired, as these improve the sensible heat storage capacity and the power density of the system (Table 2). Of these, the thermal conductivity is far easier to improve through external means such as the containers for PCMs. All ssPCMs should also have low volume change on phase transition.

High volume change can lead in time to decreased contact area between the thermal energy storage materials and the heat transfer surfaces, thus diminishing both the energy and power densities. Other problems such as leakage are also associated with this volume change. The materials should also have low vapor pressure inside the operating temperature range. While encapsulation into porous silica can control the leakage of molten PCMs, evaporation remains a potential problem, especially in the case of organic materials. High crystallization rates and low supercooling degrees are also desired, as the crystallization process is hampered due to nanoconfinement effects [63].

All phase change materials must be chemically inert inside their thermal operating range, exhibiting a low rate of thermal decomposition and insignificant reactivity towards their containers. Reactivity and corrosiveness are in general problems associated with inorganic PCMs (molten salts, salt hydrates). The materials should also possess excellent chemical stability under repeated heating-cooling cycles. Both organic and inorganic PCMs can exhibit loss of thermal storage capacity due to unwanted chemical reactions during use. The materials should ideally be safe, nontoxic, flammable or explosive. It is worth noting that impregnation into porous silica can reduce the corrosiveness, as less PCM surface area is directly exposed to the exterior. Silica is also non-toxic, non-flammable and non-explosive. However, the high specific surface area of the silica matrix can promote unwanted chemical reactions, so the long-term stability of any new shape-stabilized phase change material should be investigated [64]. An important aspect is related to the compatibility between the porous silica and the active heat storage material. The PCM should wet the silica surface and have enough surface tension to ensure the formation of shape-stabilized materials. Higher surface tension and attractive forces between the PCM molecules and silica surface can also increase the amount of impregnated heat storage material, yielding a higher storage capacity for the final composite.

Finally, economic and environmental concerns must also be taken into account. The PCMs should be inexpensive and abundant. Phase change materials should have low impact on the environment. Materials obtained from waste or renewable sources, such as paraffins or fatty acids, are especially important in this context.

The synthetic route taken to obtain porous silica-phase change composites is a critical parameter affecting the materials final properties. Two approaches are commonly used: the direct synthesis of the shape-stabilized materials and the impregnation of phase change materials into the porous silica matrices. The main advantage of the direct synthesis approach lies in its simplicity. The direct approach is often carried out as a “one-pot” sol-gel synthesis of the silica framework in the presence of the phase change molecules. Even though this method offers less control over the silica pore structure and size distribution, the direct synthesis can be employed to obtain for example PCMs encapsulated into hollow porous silica microspheres [65].

The main advantage of the impregnation approach consists in the possibility of tailoring the properties of the porous matrix before the addition of the heat storage material. This possibility is especially important in the case of organic phase change molecules containing long alkyl chains. These PCMs are hydrophobic while the silica framework is hydrophilic, leading to poor compatibility between the two components. Hydrophobic functionalization of the silica surface can be used to alleviate this concern [66].

**Figure 3:**
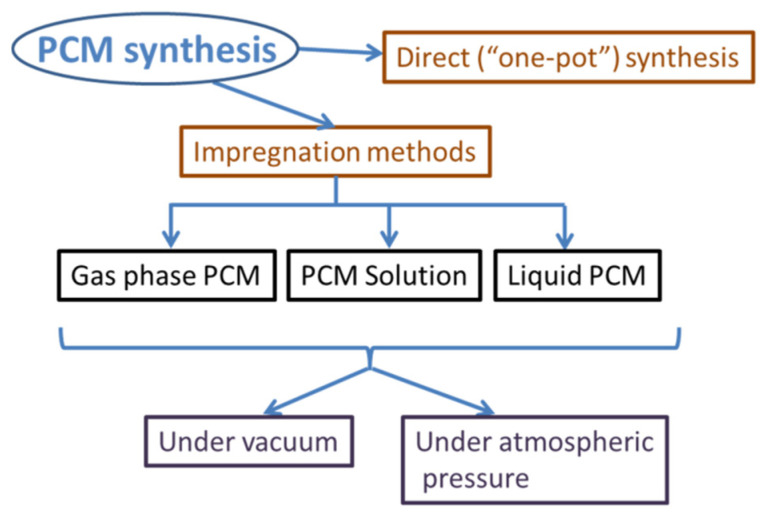
Classification of PCM synthesis methods.

The impregnation synthesis method can be further classified depending on the physical state of the phase change material. Impregnation can be carried out using a PCM solution, molten PCM or even gaseous phase change molecules (Figure 3). Solid PCM loading in typically not performed since it would require energetic methods such as grinding or ball milling, which are destructive towards the porous silica structure. The impregnation of gas-phase PCMs is rarely employed, since the organic PCMs are often constrained by low thermally stability while inorganic PCMs have low vapor pressures. Precise control over the loaded weight fraction is also difficult to achieve using this method. Furthermore, the impregnation process can be carried out under ambient atmosphere, inert gas, or vacuum (Figure 3).

The nanometer dimensions of the silica pores hinder diffusion of PCM species during the loading process and the counter diffusion of physisorbed water and air. Pristine silica surfaces are hydrophilic due to the presence of silanol groups. Thus, water molecules are readily adsorbed from the atmosphere and strongly bound to the surface, hindering the adsorption of PCM species. While vacuum and/or heat pretreatments of the silica matrices can remove the water molecules, these add additional complexity and cost for large scale applications. Solution impregnation methods require that the solvent is preferentially removed over the PCM species. Thus, the solvent must have higher vapor pressure than the active heat storage materials. Solution impregnations methods require solvent removal, so they can combine the benefits of high temperature or vacuum pretreatments.

### 2.4. Porous Silica Nanocomposites: Structure and Physico-Chemical Properties under Nanoconfinement

**Figure 4:**
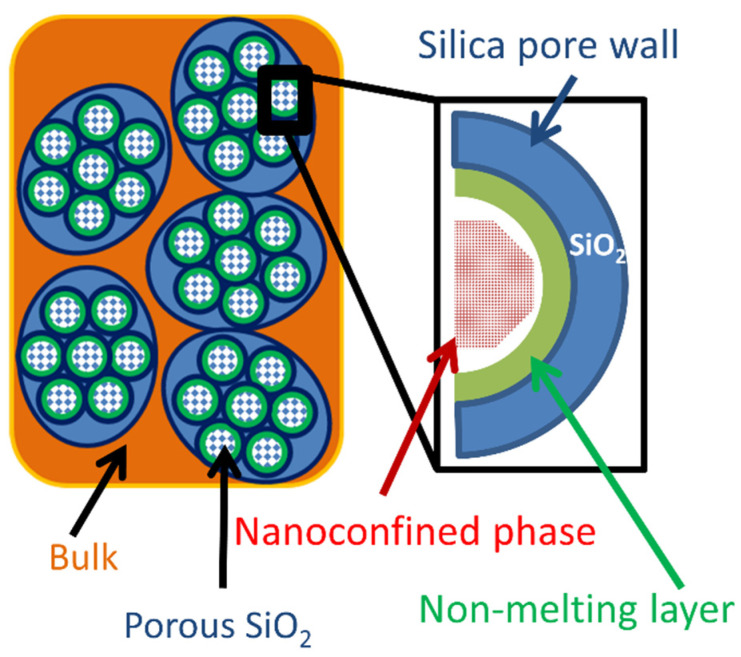
Schematic representation of porous silica composite PCMs showing the non-melting layer, bulk and nanoconfined phases.

The structure of the porous silica composites is influenced by both the synthesis method, active heat storage phase and the nature of the inorganic matrix. The active heat storage phase can be found either inside the silica pores or in the interparticle space (Figure 4). The former can be denoted as the nanoconfined phase, while the latter represents the interparticle or bulk phase [67]. The pore volume must also be able to accommodate the volume change during phase transition, as for most substances the liquid phase has a lower density than the solid. Thus, some fraction of the silica pores will be empty below the melting point of the nanoconfined PCM phase.

An important feature of nanoconfined solids inside nanometer-scale pores is the existence of an interface layer between the nanoconfined solid and the pore walls [68]. The interface layer behaves like an amorphous solid or a liquid layer and therefore it does not participate in latent heat storage through the melting-crystallization phase transitions. This layer is commonly denoted “the non-melting layer”. For example, in the case of nanoconfined water into MCM-41 it was estimated that the interface layer remains liquid down to temperatures of 21–26 K [69]. The thickness of the non-melting layer increases with increasing temperature and pore curvature [70,71]. The layer thickness is an important parameter for estimating the heat storage loss due to the non-melting interface layer. Its thickness varies between 0.3 and 2.6 nm, deepening on the nanoconfined substance [66,72]. Up to 4 monomolecular layers can be part of the interface layer in the case of small molecules such as water or carbon tetrachloride [73], while for larger molecules such as paraffins or fatty acids, the layer thickness usually corresponds to the length of one molecule [67]. Similar values are obtained even when the nanoconfined PCM is an ionic compound such as molten salts [74].

The most notable difference between a nanoconfined PCM phase and its bulk counterpart is the change in melting/crystallization temperature. The nanoconfined melting point (m.p.) is always reduced in comparison with bulk in the case of silica-based matrices [75]. The m.p. change is a consequence of reduced particle size in the nanometer range and it can be quantified using the Gibbs-Thompson equation (Equation (1)). This equation is derived from the Kelvin (Equation (3)) and Clausius-Clapeyron (Equation (4)) equations. The Equation (2) is obtained assuming that the molar volumes of the liquid and solid phases are nearly equal ($V_{m}\approx V_{l}\approx V_{s}$) and that the solid phase is wetted by its own liquid phase ($\gamma_{s}-\gamma_{l}=\gamma_{sl}$).

(1) $$T\left(\infty \right)-T\left(r\right)=\Delta T=-\frac{T\left(\infty \right)}{\Delta H_{f}}\frac{\alpha }{r}\left(V_{l}\gamma_{l}-V_{s}\gamma_{s}\right)$$

(2) $$\Delta T\approx -\frac{T\left(\infty \right)M\gamma_{sl}}{\rho \Delta H_{f}}\frac{\alpha }{r}$$

where ΔT is the difference between the melting points of a particle with radius *r*, T(r) and the bulk phase, T(∞), $\Delta H_{f}$ is the enthalpy of fusion, *V* and *γ* are the molar volume and surface tension of the liquid (l) and solid phases (s), *α* is a shape parameter denoting cylindrical or spherical pore geometry, *M* is the molar mass, *ρ* the density and $\gamma_{sl}$ denotes surface tension of the solid-liquid interface.

(3) $$ln\frac{p}{p_{0}}=\frac{2\gamma V_{m}}{rRT}$$

where *p* and *p*_0_ represents the vapor pressure over a capillary meniscus and the saturated vapor pressure, respectively, *γ* is the surface tension, $V_{m}$ is the molar volume, and *r* is the capillary radius

(4) $$\frac{dT}{dP}=T\left(\infty \right)\frac{\Delta V_{m}}{\Delta H_{f}}$$

The Gibbs-Thompson equation is derived from classical concepts and it does not consider other effects which can affect the nanoconfined melting process, such as inhomogeneity of the non-melting layer, effect of pore curvature or crystal defects. The use of this equation becomes unreliable for pores with diameters less than 5 ± 2 nm [76,77]. Furthermore, the classical Gibbs-Thompson equation does not take into consideration the existence of the non-melting interface layer. The layer thickness, *t*, can be incorporated by computing the nanoconfined particle radius as the difference between the pore size and non-melting layer thickness (Equation (5)).

(5) $$r=\frac{d_{pore}}{2}-t$$

where $d_{pore}$ is the silica pore diameter [77].

Other effects can also influence the nanoconfined melting points. For example, in closed pores the change in molar volume during phase transition can lead to a change in pressure according to the Clausius–Clapeyron equation (Equation (4)). The m.p. will decrease with increasing pressure for most substances except water. This effect is not typically considered since the porous silica matrices have open pore networks, enabling isobaric phase transitions [78].

The experimental determination of the non-melting layer thickness *t* becomes essential for quantifying the thermal behavior of the nanoconfined PCMs. The *t* parameter can be obtained by fitting the melting point depression ΔT versus the pore radius or diameter for several samples. Care must be taken to ensure that all porous silica matrices have similar pore geometry (same *α* in Equation (2)) and that the pore diameter is sufficiently large in order to minimize deviations due to high pore curvature. This can be achieved by avoiding porous matrices with pore diameters lower than 3–4 nm such as MCM-41. It is also important to note that while the Equation (2) can be fitted as a linear function of 1/*r*, doing so will lead to higher deviation for the larger *r* values, in contrast with physical reality [79].

Another approach was introduced by Lee et al., by successively evaporating part of the active heat storage compound and measuring the heat of fusion of the bulk and nanoconfined phases [80]. This allowed the construction of a heat of fusion versus weight fraction curve and the determination of the amount of substance at zero enthalpy, assumed to fully correspond to the non-melting layer.

**Figure 5:**
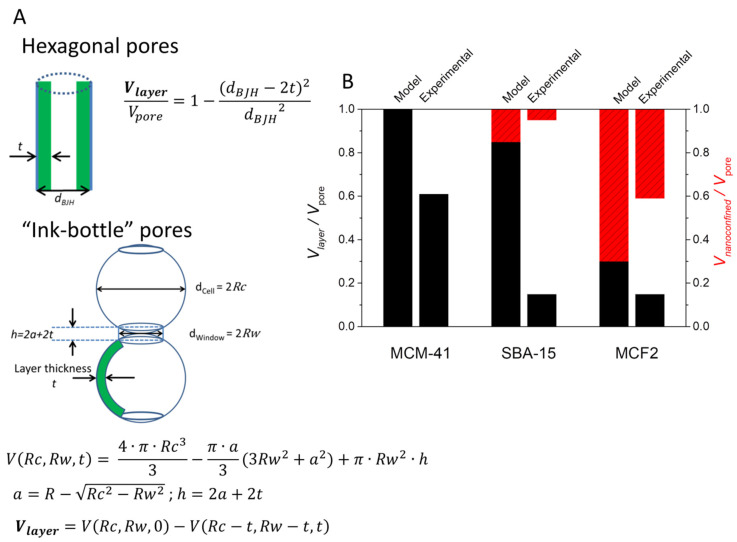
(**A**) Non-melting layer volume model based on porosity data and (**B**) comparison between the experimental and model volumes of the nanoconfined and non-melting phases in the case of NaNO_3_/KNO_3_ eutectic. Adapted with permission from ref. [74].

The volumes occupied by the nanoconfined phase, the non-melting layer and the empty pore volumes can be computed using the non-melting layer thickness and porosity data. Our group constructed a simple model using geometric considerations and assessed the phase distribution of lauric acid encapsulated into various mesoporous silica carriers [67]. The non-melting layer volume was computed as the difference between the matrix total pore volume minus the theoretical volume of a pore with a radius of *r* from Equation (5) (Figure 5A). The model could also be applied for inorganic PCMs such as eutectic sodium nitrate–potassium nitrate mixture [74]. Using the computed non-melting layer volume, the nanoconfined phase volume and empty pore volumes could be found and compared with the experimentally determined values (Figure 5B). This model was expanded by removing the geometric pore shape considerations and using the experimental pore volume versus size distribution determined from nitrogen porosimetry data [66]. The non-melting layer volume was computed as:

(6) $$V_{layer}=\underset{i}{\sum }\{\begin{array}{c} V_{pore\;}\left(i\right)\cdot \left[d{\left(i\right)}^{2}-{\left(d\left(i\right)-2t\right)}^{2}\right]/d{\left(i\right)}^{2},\;d\left(i\right)>2t\\ V_{pore\;}\left(i\right),d\left(i\right)\leq 2t\; \end{array}\;$$

where $V_{layer}$ is the non-melting layer volume, $V_{pore}\left(i\right)$ and d(i) are the pore volume and pore diameter at each data point *i* of the pore size distribution curve. Pores with diameters lower than twice the layer thickness are assumed to be completely occupied by the non-melting layer [74].

A simpler approach was employed by Liu et al., which introduced a shape parameter *N* taking values between 2 and 3 [76]. The ratio of nanoconfined phase volume ($V_{NC}$) to the total pore volume ($V_{pore}$) was computed using (Equation (7)). Nomura et al. have introduced a similar empirical formula and showed good agreement with Equation (7) for both cylindrical pores with *N* = 2 and spherical pores with *N* = 3 [81].

(7) $$\frac{V_{NC}}{V_{pore}}={\left(1-\frac{2t}{d_{pore}}\right)}^{N}$$

Regardless of the model used to quantify the volume of the non-melting layer and its influence on the total heat storage capacity of the nanocomposite PCMs, it can be seen that porous silica with lower pore sizes have a proportionally larger volume fraction occupied by this layer. Recalling that the typical layer thickness is up to ~2.5 nm, this means that common porous matrices such as mesoporous MCM-41 and SBA-15 are unsuited for being used as matrices in obtaining PCMs with high enthalpy values.

An important measure of the presence of the non-melting layer and energy storage capacity is the heat storage efficiency, *η*. The efficiency is defined as the ratio of experimentally determined heat of fusion ($\Delta H_{composite}$) to the theoretical heat of fusion of an equivalent amount of PCM as that present in the composite (Equation (8)).

(8) $$\eta \left(\%\right)=100\;\cdot \;\frac{\Delta H_{composite}}{w_{PCM}\Delta H_{PCM}}$$

where $w_{PCM}$ is the weight fraction of the PCM and $\Delta H_{PCM}$ is the heat of fusion of the pure PCM [67].

## 3. Phase Change Materials Containing Porous Silica Matrices and Different Heat Storage Compounds

The main organic phase change materials consist of paraffins, fatty acid, and their derivatives, sugar alcohols and polymers, while the principal inorganic PCMs are salt hydrates, molten salts, and elemental compounds. The most used porous silica matrices include aerogels and xerogels, mesoporous silica and silicates, silica nanoparticles either as-prepared or fused. A large variety of possible nanocomposites can be obtained, enabling control over the thermal and structural properties. Additional materials are sometimes added to improve the properties of the resulting samples. The most common additives are carbon-based materials, which have high thermal conductivity. The recent progress in the field of porous silica-based phase change materials is presented in the following subchapters, based on the main type of PCM used to obtain the nanocomposites.

### 3.1. Paraffins

Paraffins or paraffin waxes are mainly composed of long chained *n*-alkanes. Although flammable, they are relatively unreactive. While pure compounds are sometimes used, most often paraffins are mixtures. A high number of paraffins are commercially available, covering a large temperature range in terms of their melting points. They also have high heat of fusion values, often exceeding 200 J g^−1^. The long alkyl chains have a parallel arrangement in solid state, which requires a high amount of energy to break, thus yielding high heat of fusion values. *N*-alkanes sometimes exhibit a secondary solid-solid phase transition below their melting point. This is an order-disorder transition, caused by a partial destruction of the parallel chain structure. Paraffins are a by-product of petroleum refining, making then attractive from the standpoint of waste reutilization.

Paraffin–porous silica composites can be obtained through direct sol-gel synthesis of the silica matrix in the presence of paraffin, a suitable structure directing agent, and sometimes an emulsifier [82,83,84,85]. Cetyltrimethyl ammonium bromide (CTAB) and *n*-pentanol as emulsifier were found to give superior results over sodium dodecyl sulfate (SDS) or Span 80 and Tween 80 [82]. A composite with a heat storage capacity of 95 J g^−1^ and a m.p. of 30 °C was obtained when starting from a paraffin wax with a Δ*H_f_* value of 142 J g^−1^ and similar m.p (Table 3). Simple hydrolysis of tetraethyl orthosilicate in acid medium could yield a composite with up to 92% wt. paraffin, although it is unlikely such a material also had shape-stability [83]. Similarly, paraffin with 110 °C m.p. was encapsulated into silica at a 1:1 wt. ratio of paraffin to TEOS [84]. The material was added in-situ for providing thermal control of the methyl methacrylate polymerization reaction. An optimization of the sol-gel synthesis using sodium silicate in terms of pH, temperature, paraffin/water and paraffin/silica was carried out [86,87]. Shape-stabilized composites could be obtained at pH = 4.5 and 35 °C. Nanoencapsulated *n*-octadecane into 170–560 nm silica shells shows a remarkable dependence of the melting point decrease with particle size [88]. The m.p. decreased varies from 2.2 °C for the 170 nm nanoparticles to 1.2 °C for the 560 silica NPs. C17-C20 *n*-alkanes, sodium silicate and Pluronic P104 were used to create shape-stabilized PCMs through direct sol-gel synthesis [89]. Around 45–55% wt. *n*-alkanes were encapsulated, while the composite melting enthalpy varied between 61–81 J g^−1^ depending on the hydrocarbon chain length. The composites had increased thermal stability with respect to pure paraffins and good reliability after 100 heating-cooling cycles.

Liu et al. designed mesoporous silica nanospheres and nanocapsules for *n*-eicosane through direct sol-gel synthesis [90]. The matrices have pore diameters of 7.9 and 3.3 nm and could be used to obtain shape-stabilized PCMs with 122 and 113 J g^−1^ heat of fusion values for the nanospheres and nanocapsules, respectively. The thermal conductivity increased from 0.15 for the pure paraffin to 1.17 W m^−1^ K^−1^ for the nanocapsules.

**Figure 6:**
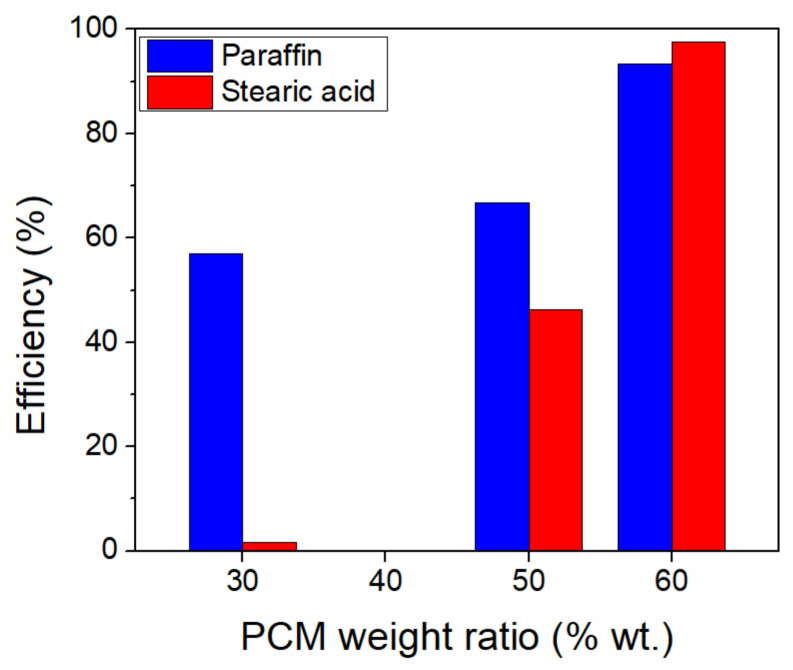
Comparison of the heat storage efficiency of paraffin and stearic acid-silica composites obtained through one-pot sol-gel synthesis. Data from ref. [85].

A comparison of the initial amount of various PCMs in the one-pot sol-gel synthesis of silica PCMs was carried out using paraffin, stearic acid and polyethylene glycol (PEG) at 30, 50 and 60% wt. ratios [85]. No heat storage capacity was obtained for any PEG-based PCMs, while a significant loss of thermal energy storage at low PCM ratios, especially for the stearic acid was noticed (Figure 6). The reduction in heat storage was correlated with the presence of nanoconfined PCMs into the silica mesopores. The thermal energy efficiency can be computed as the ratio of effective PCM content calculated based on the experimental heat of fusion values divided by the actual PCM content (Figure 6).

Impregnation of molten paraffins into an already prepared porous matrix is also used to create shape-stabilized PCMs [27,91,92,93,94]. This method offers better control over the silica pore shape, size and volume as well as paraffin mass fraction, at the cost of a two-step synthesis. Porous silica aerogels obtained by supercritical ethanol extraction were impregnated with a paraffin with a 56–58 °C melting range [27]. The most porous aerogel, with an average pore diameter of 56 nm and total pore volume of 5.22 cm^3^ g^−1^, yielded a composite with 75% paraffin. Vacuum melt impregnation was used to create expanded perlite-paraffin shape-stabilized composites, which were then coated with colloidal silica and acrylic and used to create energy storage panels for buildings [91]. Larger, 1 mm expanded perlite particles were found to have lower leakage amounts than smaller, 60 μm particles in a later study [95]. Paraffin with 28 °C m.p. was melt impregnated into raw diatomite and mesoporous MCM-41 silica [92]. Up to 55% and 60% wt. heat storage compound could be impregnated into diatomite and MCM-41, respectively. The m.p. was decreased with 2.5–3.5 °C with respect to bulk, indicating interparticle nanoconfinement effects. Vacuum melt impregnation into a silica aerogel-polytetrafluoroetheylene (PTFE) thin film achieved 62.8% wt. paraffin loading [96]. The experimental heat of fusion value was lower than the expected value (η = 83.9%), indicating the formation of a non-melting layer or an amorphous phase. The optical transmittance of the PCM thin film exhibited both temperature and wavelength dependence. Fumed silica-paraffin nanocomposites were added up to 30% wt. into cement formulations for building passive energy storage applications [93]. A shape-stabilized paraffin/fumed silica sample was obtained at 45% wt. loading and it exhibit 100% heat storage efficiency. Increasing the mass fraction of the composite PCM into the cement mixture led to a decrease of thermal conductivity from 0.127 W m^−1^ K^−1^ to 0.101 W m^−1^ K^−1^ at 30% wt. composite. Further studies showed that the addition of 3% silica nanoparticles and PCMs (paraffin or *n*-octadecane) to cement mixtures led to better mechanical properties and low chemical shrinkage [97]. A kinetic study of paraffin melt impregnation into silica aerogel showed that a maximum loading of 75% wt. can be achieved after 3 min, with little further improvements [94]. Melt impregnation of octadecane and a complex mixture of fatty acids into micronized silica nanoparticles have resulted in composites with similar melting points and reduced heat storage efficiencies [98].

A comparative study of vacuum melt impregnation into mesoporous silica, expanded graphite (EG), bentonite, diatomaceous earth and zeolite Y was carried out using hexadecane, octadecane, capric and lauric acid eutectic mixture and butyl stearate [99]. The highest hexadecane loading was obtained in the case of the mesoporous silica matrix, at 81% wt. EG exhibited PCM loading of 77% wt., with the rest of the matrices loading less than 50% PCM. The reliability of the best two matrices was tested up to 450 heating–cooling cycles at three initial PCM to matrix rates. It was found that all samples stabilize after 300 cycles. Heat of fusion vales around 100 J g^−1^ could be obtained for starting PCM: matrix ratio of 2:1 and 3:1. Nomura et al. studied the influence of varying the pore diameter of mesoporous silica between 11 and 50 nm on the thermophysical properties of the resulting octadecane-silica nanocomposites [81]. The authors found that pore radius more than 20 nm are required for the nanocomposites to retain at least 80% of their theoretical heat of fusion. Decreasing pore diameters below 40 nm leads to fast reduction in heat storage due to the increased volume occupied by the non-melting layer, as well as a proportional reduction of m.p. following the Gibbs-Thompson equation.

Solution impregnation methods can also be used to obtain paraffin–porous silica nanocomposites [100]. For example, ASTM D 87 paraffin wax was impregnated into two types of Stöber silica nanoparticles from a 1:1 (*v*/*v*) ethanol/hexane solution in the presence of polyvinyl pyrrolidone (PVP) at different mass fractions [100]. Significant paraffin loading was achieved only when using more than a 2:1 wt. paraffin to silica ratio in the initial synthesis. No supercooling was noticed, while a slight decrease in heat of fusion values indicated the presence of a non-melting layer.

A comparative study of melt impregnation, solution adsorption followed by filtration or evaporation was carried out using *n*-eicosane and silica aerogel (Enova aerogel particles IC3120, Cabot Corporation), with heptane as the solvent [101]. The highest eicosane adsorption of 84.3% wt. was obtained for melt impregnation, with the solvent methods yielding 72–75% wt. paraffin loadings. Thermal energy storage was proportional to the paraffin mass loading. The particles could be used for coating textiles to provide thermal comfort in the 35–39 °C temperature range.

The hydrophobic nature of the paraffins contrasts with the hydrophilic silica surface containing surface silanol (Si-OH) groups. One of the earliest examples in modifying the silica surface with hydrophobic moieties is the study of Li et al., which used hydrophobic fumed silica to decrease paraffin leakage from a 30:70 wt. PCM: cement formulation [102]. Leakage was eliminated when 9% wt. hydrophobic fumed silica was used with respect to pure paraffin. A follow-up study also included hydrophilic and hydrophobic silica aerogels and commercial RT21 paraffin [103]. The hydrophilic and hydrophobic silica aerogels were found to possess higher adsorption capacity for the paraffin, at 78 and 75% wt., respectively. The lower adsorption capacity of the hydrophobic aerogel could be caused by lower pore volume due to its modification. Flexible monolithic aerogels functionalized with methyl groups were obtained and used for paraffin impregnation from ethanol solution followed by evaporation [104]. Shape-stabilized samples were obtained at 70% wt. paraffin loading. The presence of a non-melting layer was assessed by the lower heat storage efficiency of the prepared composite. Similarly, octadecane, octadecanol, and stearic acid were also impregnated at 70% wt. loadings and shown to yield shape-stabilized composites with high heat of fusion values, between 127 and 141 J g^−1^ [104]. A mesoporous silica aerogel was obtained and functionalized post-synthesis through alkylation with methyl groups or by hydroxylation [105]. Paraffin wax was loaded through vacuum melt impregnation. The paraffin mass fraction was dependent on both pore volume and surface functionalization. Pore filling values below 100% were noticed for hydroxyl groups, while complete filling was achieved for methyl functionalization. Interestingly, a value of 168% pore filling was obtained for dual methyl and hydroxyl functionalization, at a paraffin loading of 88% wt., indicating adsorption in interparticle spaces [105]. A comparative study of hydrophilic or methyl-functionalized aerogel melt impregnated with paraffin RT60 was also carried out [106]. The methyl-functionalized aerogel yielded composites containing 80% wt. paraffin and 180 J g^−1^ heat storage capacities. A hierarchically porous silica monolith was functionalized with alkyl chains of varying length and used to adsorb octadecane and stearic acid [107]. The functionalized matrix presented a nanoconfined phase melting with higher enthalpy for the octadecyl functionalization than for octyl moieties. This small increase could be explained by cooperative interactions between the alkyl chains of the PCM and the silica surface functionalization, especially if both had the same length (e.g., C18 chains). Dodecane was adsorbed at 75 and 85% wt. loading into hydrophobic fumed silica [33]. The composite containing 75% paraffin exhibited shape-stabilization, while some leakage was noticed at higher loadings. On the other hand, the thermal conductivity increased proportional to dodecane loading. A similar result was obtained using RT28 paraffin and hydrophobic fumed silica for Li-ion battery thermal protection applications [108]. While composites with 70% paraffin exhibited shape-stability, leakage was noticed at 80% wt. loading.

**Figure 7:**
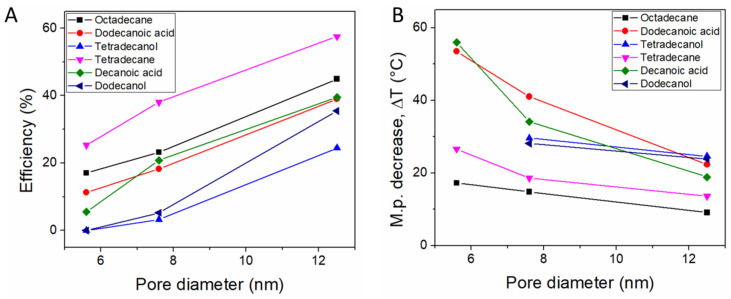
(**A**) Heat storage efficiency and (**B**) m.p. decrease versus SBA-15 mesopore diameter. Data from ref. [109].

Gas phase synthesis of composite PCMs was carried out by Choi et al., using mesoporous SBA-15 matrices with hexagonally ordered cylindrical mesopores [109]. The solid PCM was heated under vacuum in the presence of the silica matrices having pore diameters ranging from 5.6 to 12.5 nm. Octadecane and tetradecane were used as paraffins, while dodecanoic acid, decanoic acid, tetradecanol, and dodecanol were also employed. The pore fill ratio decreased from ~100% for the 5.6 nm SBA-15 to 65% for the 12.5 nm silica matrix. The authors presented evidence for both a decrease of the nanoconfined PCM melting point following the Gibbs-Thompson equation as well as for the existence of the non-melting interface layer, which caused a decrease of the recovered heat of fusion and better fit for the nanoconfined m.p. decrease (Figure 7). A subsequent study used docosane (C24 *n*-alkane) impregnated by the same method into SBA-15 mesoporous silica, resulting in a composite PCM with 53.3 J g^−1^ heat of fusion at 31 °C. [110] The composite was then theoretically investigated for dehumidifying in a fixed-bed adsorption process and it was found to have high performance due to the ability to recover heat during the first 5 min of the process.

**Figure 8:**
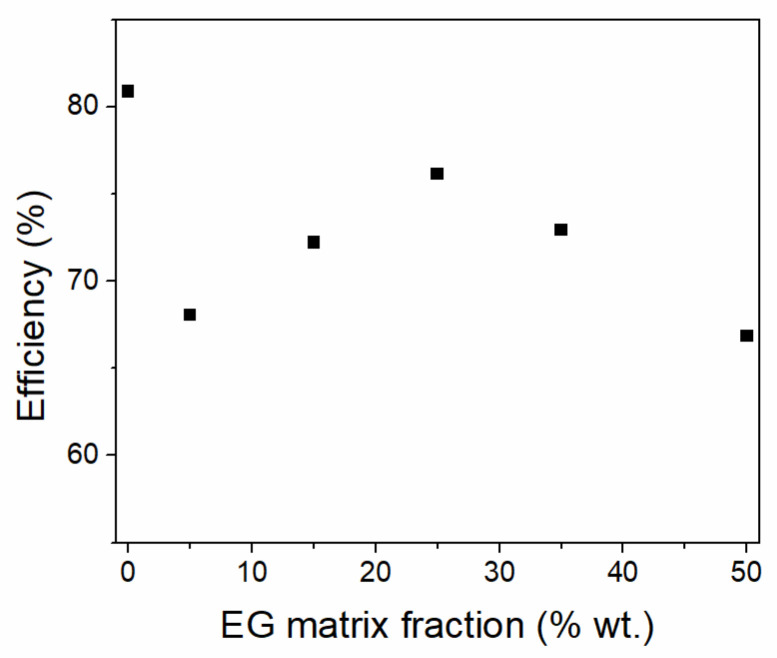
Correlation between EG weight fraction in the porous matrix and heat storage efficiency. Data from ref. [115].

The addition of carbon-based materials has been investigated for increasing the thermal conductivity of porous silica–paraffin composites [111]. Expanded graphite (EG)-silica-paraffin composites were obtained through one-pot sol-gel synthesis [111]. The composite had double the pure paraffin thermal conductivity, as well as high (104.4 J g^−1^) heat storage capacity at 27.7 °C. The influence of paraffin mass fraction was investigated in a latter work. Composites containing 2% expanded graphite, 20 nm silica nanoparticles and between 86–92% wt. paraffin were obtained through the addition of the components to melted paraffin [112]. All samples containing at most 90% wt. paraffin presented shape stability. The thermal stability, conductivity, and drop point all increased with decreasing paraffin content. Low-density polyethylene (LDPE) was also added during the sequential melt synthesis of an EG/SiO_2_/LDPE/Paraffin composite containing 30% wt. LDPE, 7% wt. EG and 3–7% wt. silica nanoparticles [113]. The 5.5% wt. silica composite showed the least leakage and was further tested for Li-ion battery thermal protection. *N*-eicosane was loaded into 20 nm SiO_2_ nanoparticles (NPs) from ethanol solution followed by evaporation, at different loadings between 65 and 75% wt. [114]. Shape-stabilized composites were obtained up to 70% wt. loading. Additional samples containing 3, 5, to 7% wt. EG were similarly prepared at a 7:3 eicosane: silica mass ratio. The thermal conductivity increased proportionally with the EG fraction, being 2.3 higher than pristine eicosane at 7% wt. EG. Porous matrices were also developed from silica gel industrial waste, which was hydrolyzed to sodium silicate [115]. Paraffin wax was vacuum melt impregnated at 80% wt. loading, while the matrix composition was varied between 0 and 50% wt. EG. The thermal conductivity of the composites increased linearly with EG content. All samples exhibit shape-stability and reliability after 1000 heating-cooling cycles [115]. While the experimental heats of fusion are lower than the theoretical value, indicating the presence of a non-melting layer, there is no linear correlation between experimental heat of fusion and EG weight fraction (Figure 8). It is worth noting that simulations of heptadecane encapsulated into MCM-41 have shown a two-fold increase in thermal conductivity over pristine paraffin, without the addition of carbon-based materials [116]. Moreover, the thermal conductivity was shown to increase with increasing PCM amount up to 70%.

Fumed silica and graphite powders were used to encapsulate paraffins for the addition into cement [117]. The obtained “thermocrete” had a latent heat storage capacity of 12.6 J g^−1^, with a melting point of 32.9 °C and a freezing point of 17.1 °C.

Graphene oxide (GO) was added during the synthesis of a paraffin–silica composite with the goal of increasing thermal conductivity [118]. The synthesis was carried out in the presence of polyvinyl alcohol (PVA) and mixed Span80 and Tween80 surfactants. The thermal conductivity of the composite is significantly increased to 1.16 W m^−1^ K^−1^ in comparison with pristine paraffin, at around 0.34 W m^−1^ K^−1^. A similar approach consisted of adding GO to molten paraffin in the presence of a surfactant followed by porous silica synthesis through interfacial polycondensation in an oil–water emulsion [119]. Then, 5% wt. of the paraffin-GO-SiO_2_ material was added to PVC formulations, which showed improved thermal conductivity with respect to pristine PVC.

Other functionalities can also be added to the composite PCMs. For example, magnetic response coupled with thermal storage was achieved by adding Fe_3_O_4_ nanoparticles to a *n*-eicosane (C_20_H_42_) suspension, followed by silica hydrolysis and condensation in the presence of CTAB, which resulted in 4–6 μm core-shell particles [120]. Materials with up to 70% wt. PCM versus SiO_2_ were obtained, possessing the corresponding heat of fusion based on the paraffin weight fraction (Table 3). Ti_4_O_7_ was added to paraffin-silica nanocomposites for solar energy adsorption [121]. A 3.3-fold increase in thermal conductivity with respect to pristine paraffin was also achieved for the SiO_2_-Ti_4_O_7_ matrix.

**Table 3:** Representative porous silica–paraffin nanocomposites.

PCM			Porous Silica Composite				Ref.
Sample	m.p. (°C)	Δ*H_f_* (J g^−1^)	Sample/Synthesis	%PCM (wt.) *	m.p. (°C)	Δ*H_f_* (J g^−1^)	
Paraffin wax	29.0	142.0	Direct synthesis/CTAB + *n*-pentanol	-	30.0	95.0	[82]
Paraffin	51.0	151.5	Direct synthesis/TEOS/HCl	92.1	50.2	139.6	[83]
Paraffin	57.0	-	Aerogel/supercritical EtOH	75	-	-	[27]
Rubitherm RT 28	-	-	TEOS/HCl, EG, paraffin	-	27.7	104.4	[111]
*n*-eicosane	39.2	237.1	*n*-C_20_H_42_,Fe_3_O_4_@SiO_2_	70	39.2	170.2	[120]
PX25	~23	96	9% hydrophobic fumed silica vs. PCM; 30:70 wt. composite: cement	27.3	~23	14.2	[102]
Paraffin	50.1	173.9	Parafin@SiO_2_-GO/PVA, Span80, Tween80	49	49.7	87.1	[118]
25# Paraffin	25.8	107.6	Vacuum melt impregnation	-	21.6	56.3	[91]
*n*-eicosane	37.13	249.0	Aerogel melt impregnation	84.3	36.8	198.4	[101]
Paraffin	57.7	161.4	TEOS/HCl	60	58.2	98.0	[85]
Paraffin	51.9	184.1	2% EG; 8% 20 nm SiO_2_; melt addition	90	51.8	168.3	[112]
Octadecane	26.5	2300	Gas transport/12.5 nm SBA-15	-	17.4	103.3	[109]
Tetradecane	6.2	216.0		-	−7.4	124.2	
Paraffin	49.7	200.4	Solvent impregnation/SiO_2_ NPs	80	52.0	156.6	[100]
Paraffin	28.0	168.0	Melt impregnation/MCM-41	60	25.5	95.0	[92]
Paraffin	42.2	243.0	Vacuum melt/SiO_2_-PTFE aerogel	62.8	42.0	128.0	[96]
Paraffin	59.6	191.1	Solution impregnation/CH_3_-fucntionalized aerogel	70	59.6	112.9	[104]
*n*-Eicosane	36.9	243.3	Solution impregnation/7% EG/70:30 Eicosane:20 nm SiO_2_ NP	65.1	35.4	135.8	[114]
Paraffin wax	63.8	209.1	Vacuum melt/CH_3_/HO-aerogel	88	63.7	163.6	[105]
Paraffin wax	25.7	198.0	Vacuum melt/SiO2-EG 1:1	80	26.7	105.9	[115]
Paraffin	56.8	182.2	Melt impregnation/aerogel	75	56.3	165.2	[94]
Hexadecane	17.7	220	Vacuum melt/mesoporous silica/300 heat-cool cycles	45	17.1	100.1	[99]
Octadecane	29.9	223	Vacuum melt/mesoporous silica	45.3	28.9	84.5	[81]
Octadecane	28.5	212.6	Direct synthesis	-	26.3	99.3	[88]
Nonadecane	29.4	201.0	Direct synthesis	-	26.2	80.8	[89]
Octadecane	28.2	232.5	Vacuum melt/SiO_2_ NP	70	27.7	85.0	[122]

* “-“ = not available.

### 3.2. Fatty Acids and Derivatives

Fatty acids are naturally occurring carboxylic acids containing a long *n*-alkane or alkene chain. The high melting enthalpy of these compounds arises from the attractive interactions between the log chains as well as from hydrogen bonding between the carboxylic groups. Fatty acids are especially interesting as a source of renewable, green phase change materials. Similar compounds containing long alkane chains and hydrophilic groups (hydroxyl, amine, ester, amide etc.) can also be used as heat storage materials. Unlike paraffins, fatty acids and their derivatives present both hydrophobic and hydrophilic moieties, which enable additional pathways to tailor their adsorption and properties through functionalization of the porous silica matrices.

The direct sol-gel synthesis in the presence of fatty acids can be used to obtain porous silica composites with xerogel silica framework [123]. Up to 75% wt. capric acid (CA)-palmitic acid (PA) eutectic yielded shape-stabilized PCMs through this method. While the composite exhibited 103.4 J g^−1^ heat storage, its thermal conductivity was decreased. Exfoliated graphite nanoplatelets could be added up to 6% wt. in order to increase the thermal conductivity and simultaneously increase the fatty acid loading up to 85% wt. Up to 65% wt. lauric acid (LA) could also be encapsulated through direct synthesis [124]. The composite m.p. was decreased by 2 °C with respect to bulk LA, indicating nanoconfinement effects. Careful selection of the synthesis method can result in the formation of core-shell particles. For example, PA microcapsules were obtained using SDS as surfactant [125]. The acid m.p. was decreased by 1–2 °C with respect to bulk, while up to 89% heat of fusion versus bulk was obtained. A 72/28 capric-myristic acid eutectic was encapsulated into silica through direct sol-gel synthesis [126]. The composite containing 40% fatty acids maintained its enthalpy and m.p. after 200 heating-cooling cycles. The direct sol-gel synthesis of stearic acid (SA)-silica composites showed increased leakage and mass loss for samples containing more than 76% wt. SA [127]. Interestingly, all samples had lower m.p. and enthalpy values than expected on the basis of SA mass, indicating the presence of a non-melting layer for this type of materials. Shape-stabilized PCMs containing up to 70% wt. 1, 8-octanediol (ODL) were also prepared by the sol-gel method [128]. While the m.p. decreased by around 1 °C in comparison with bulk ODL, the enthalpy was close to the value expected based on the mass fraction of the diol (Table 4).

The influence of methyl groups on lauric acid composite PCMs obtained through direct sol-gel synthesis was investigated using methyl triethoxysilane (MTES) as the organic group source [129]. MTES to TEOS weight ratios of 0.45 to 0.53 yield composites with the best thermal energy storage properties as well as hydrophobicity (water contact angle of 114–125°) which could enable these materials to store heat in humid environments.

**Figure 9:**
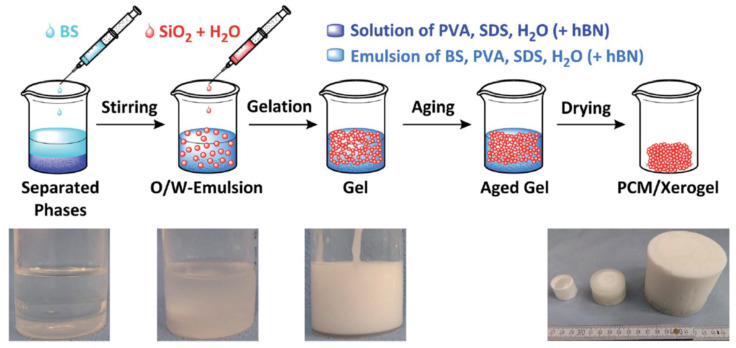
Schematic representation of butyl stearate monolith synthesis. Reproduced from [130]. Published by The Royal Society of Chemistry, under CC BY-NC 3.0 license.

Marske et al. obtained a porous silica monolith containing butyl stearate (BS) using sol-gel synthesis in the presence of SDS and PVA surfactants [130]. Monolithic and form-stable composites were obtained to a BS loading of 84% wt. (Figure 9). The 84% BS sample had a compressive strength of 0.7 MPa at 30 °C and higher thermal conductivity than pure BS (0.22 versus 0.12 W m^−1^ K^−1^). The m.p. of the composite was increased by 2.4 °C with respect to BS, which was explained as arising from the pressure increase in closed pores following the volume change during the phase transition. Heat of fusion values of 80.4 J g^−1^ remained unchanged before and after 6000 heating-cooling cycles. The composite monoliths contained both mesopores in the 2–50 nm range and macropores up to 6 μm.

Impregnation of molten fatty acids or derivatives directly into the porous silica matrices is a promising method for obtaining ssPCMs. A ternary eutectic consisting of lauric (LA), capric (CA) and palmitic (PA) acids (CA:LA:PA = 61.9:31.0:7.1) was prepared and used to create composites PCMs using electrospun silica mats as the matrix [131]. The fibers annealed at 600 °C could adsorb 81% wt. eutectic (Table 4), with no loss of heat storage efficiency. In a follow-up study, melt impregnation of a quinary fatty acid eutectic was carried out for 10h using electrospun silica nanofibrous mats as the matrix [132]. The flexible composites exhibited up to 92.2% wt. loading capacity, with shape-stability at 80.2% wt. loading. A ternary eutectic consisting of lauric acid (LA), palmitic acid and paraffin (PAR) with m.p. = 58 °C was prepared in a LA:PA:PAR = 54.25:15.75:30 ratio. The eutectic was then melt impregnated into a disk containing silica nanoparticles obtained from spent lead-acid batteries mixed with high density polyethylene (HDPE) [133]. The weight fraction of the remaining eutectic after leakage test was proportional with the SiO_2_ NPs content, decreasing from 75 to 38% wt. when the SiO_2_ fraction decreased from 100% to 0%. Good heat of fusion values were obtained, as well as increased thermal conductivity proportional to the HDPE content [133].

Melt impregnation of ternary CA:PA: SA = 79.3:14.7:6.0 eutectic into silica nanoparticles was used to prepare composite PCMs for building energy savings applications [134]. A maximum eutectic loading 75% wt. could be achieved. The composite has good reliability, with only 5% loss in enthalpy after 500 heating–cooling cycles. A commercial fatty ester was impregnated into 16 μm silica gel particles for perishable food storage applications [135]. While the resulting composite had a PCM loading of 55% wt., the enthalpy only corresponded to 36% of pure fatty ester. Molten caprylic acid was mixed with six types of porous supports (bentonite, natural clay, diatomaceous earth, expanded pearlite, silica gel and activated charcoal) in order to obtain shape-stabilized PCMs [136]. Expanded pearlite had the highest fatty acid loading at 59% wt., while silica gel incorporated 48% PCM. The heat storage efficiency of the composites was reduced for all samples except expanded pearlite and activated charcoal.

Gas phase transport of lauric acid, capric acid, tetradecanol and dodecanol was investigated using three types of mesoporous SBA-15 with increasing pore diameters from 5.6 nm to 12.5 nm [109]. Nanoconfined m.p. decrease and non-melting layer formation were noticed.

Fumed silica was impregnated with solution of stearic acid (SA) in chloroform, followed by vacuum distillation [137]. The SA m.p. was decreased with 0.9 °C with respect to bulk and the amount of acid was computed solely on the basis of heat of fusion (Table 4). The composite exhibited high thermal reliability and it retained the same heat storage capacity after 600 heating–cooling cycles. Tannic acid templated mesoporous silica having an average pore diameter of 8.4 nm was loaded with stearic acid from ethanol solution at 50%, 60% and 70% wt. [138]. The 50% loaded sample exhibited only small heat of fusion, indicating that most of the acid is present in the non-melting layer. The 70% wt. loaded sample exhibited two melting events, corresponding to the crystalline nanoconfined and interparticle phases.

Using sacrificial polystyrene nanoparticles, Fan et al. prepared hollow mesoporous microspheres 500 nm in diameter and used them as carrier for stearic acid [139]. The hollow microspheres were loaded up to 70% wt. with SA. Interestingly, the supercooling exhibited by the fatty acid was reduced, with lower loading yielding lower supercooling. A mesostructured onion-like silica (MOS) material, having large spherical pores up to 50 nm was also used to create stearic acid composite PCMs through ethanol solution impregnation [140]. 70% wt. was found to be the maximum loading without leakage. A 50% wt. SA loading yielded no heat of fusion, indicating the formation of a non-melting layer. The 70% SA-MOS composite exhibited good reliability after 50 heating-cooling cycles.

**Figure 10:**
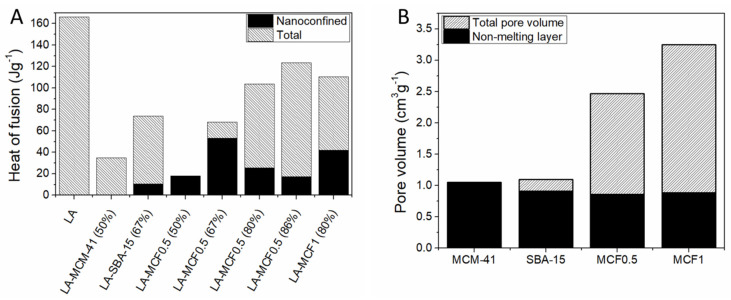
(**A**) Heat of fusion corresponding to the nanoconfined phase and total enthalpy and (**B**) non-melting layer specific volume as a fraction of total pore volume for four types of MSN matrices. Data from ref. [67].

The effect of lauric acid (LA) solution impregnation onto mesoporous silica matrices with varying pore diameters and volumes at different mass fractions was investigated by our group [67]. MCM-41 and SBA-15 having hexagonally ordered mesopores of 2.7 nm and 6.3 nm and two types of mesocellular foam silica (MCF) with “ink-bottle” pores up to 34.9 nm were used as matrices. LA to silica mass fractions from 1:1 to 6:1 were investigated. Up to 83% wt. LA could be loaded into the 34.9 nm MCF and still retain shape-stability. No heat of fusion was noticed at 1:1 wt. ratio indicating the presence of a significant non-melting layer. All samples containing more than 50% wt. LA showed two melting and two crystallization processes, indicating the presence of both nanoconfined and interparticle phases of the fatty acid. The ratio of the enthalpy of the two processes decreasing with increasing LA fraction, confirming that the first effect is caused by nanoconfinement inside the silica pores (Figure 10A). Using Equations (2) and (5), the non-melting layer thickness was estimated at 1.8 nm, corresponding to the length of one fatty acid molecule. Based on the thickness, the theoretical non-melting layer volume was computed (Figure 10A). The difference between the heat of fusion expected based on the LA fraction and the experimentally determined value was attributed to LA molecules present in the non-melting layer (Figure 10B). The results show that mesoporous silica with average pore diameters less than 20 nm are unsuited for PCM applications, since most of the pore volume will be taken by the non-melting layer. Even for MCF1 with a maximum pore diameter of 34.9 nm, 27% of the total pore volume corresponds to this layer. The impregnation of lauric acid (LA) into mesocellular foam silica (MCF0.5) at 40, 50, and 60% wt. in a different study also showed that no heat of fusion was detected at 40% wt. loading, indicating the formation of the non-melting layer [141].

A similar study was performed in the case of adipic acid, at three (50, 60, 67%) loading and using MCM-41, SBA-15 and three MCF matrices. [76] The melting point of the nanoconfined phase was decreased in accordance with Gibbs-Thompson equation. Higher fatty acid loading also influenced the m.p. indicating incomplete mesopore loading, especially for the MCF-type matrices. The adipic acid nanoconfinement was also shown to increase supercooling up to 33 °C for the nanoconfined phase and 15 °C for the interparticle fatty acid phase.

**Figure 11:**
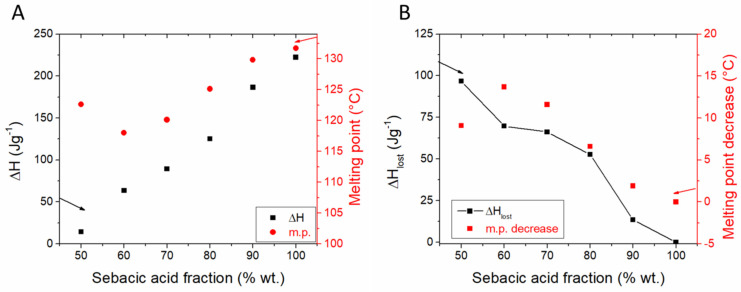
(**A**) The heat of fusion and m.p. and (**B**) lost enthalpy and m.p. decrease of sebacic acid-MCM-41 composite PCMs. Data from ref. [143].

Myristic acid (MA) was loaded through solution impregnation followed by vacuum drying into wrinkled mesoporous silica (WMSN), consisting of radially ordered mesopores which increase in diameter from the core to the shell [142]. 65% wt. fatty acid composites showed no leakage on a filter paper and good reliability after 50 heating-cooling cycles. The thermal conductivity of the 65% wt. MA composite also increased from 0.27 to 0.37 W m^−1^ K^−1^ in comparison with the pure fatty acid. The influence of the sebacic acid fraction in composites containing MCM-41 prepared by solution impregnation was tested between 50 and 90% wt. [143]. Composites with less than 80% fatty acid exhibited shape stabilization. All samples have lower heat of fusion and m.p. values than expected based on the mass fraction of the acid (Figure 11A). The lost enthalpy is proportional to the MCM-41 content (Figure 11), while the lowest m.p. was found for the 60% wt. sample. These results indicate that the non-melting layer occupies a significant pore volume and the increasing m.p. can most easily explained as interparticle nanoconfinement due to the lack of two distinct melting events.

Stearic acid, octadecane and octadecanol were used to prepare shape-stabilized PCMs through vacuum melt impregnation using ~200 nm silica nanoparticles [122]. Shape-stabilized materials with up to 70% wt. PCM could be obtained. No significant melting enthalpy was recovered for PCM loading under 40% wt. for stearic acid and octadecane or 60% for octadecanol. The higher non-melting layer fraction in the case of octadecanol was explained as arising from supramolecular hydrogen bonding between the PCM molecules and the silanol groups present on the nanoparticle surface.

The functionalization of the porous silica surface with various organic groups can affect the properties of the resulting composites, since fatty acids and their derivatives are hydrophobic while the silica surface contains hydrophilic silanol groups. Octadecyl functionalized hierarchically porous monolith containing both ~15 nm mesopores and macropores larger than 1 μm showed the appearance of a nanoconfined stearic acid phase [107]. Hexamethyldisilazane (HMDS) was employed for creating methyl-functionalized aerogels [144]. The functionalized aerogels were loaded with palmitic acid or octadecanol through vacuum melt impregnation and compared with pristine aerogels. The methylation treatment increased the pore volume of the aerogel from 3.35 to 4.40 cm^3^ g^−1^ and average pore diameter from 11.2 to 12.6 nm. The methyl functionalization increased the PCM adsorption capacity with 10–20% wt. and the heat of fusion values with 30–45 J g^−1^ in comparison with pristine matrices. The increase could be explained by a synergistic effect of increasing pore volume and diameter coupled with stronger intermolecular interactions between the aerogel pore surface and the hydrophobic PCM molecules.

**Figure 12:**
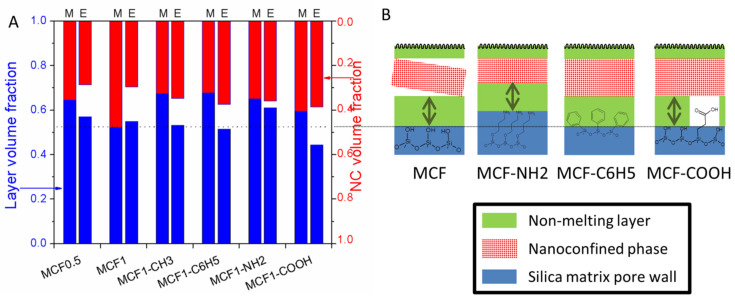
(**A**) Modeled (M) and experimental (E) volume fractions of the non-melting layer, nanoconfined SA and empty pores and (**B**) schematic representation of the pristine and functionalized pore composition. Adapted with permission. Ref. [66]. Copyright Elsevier B.V. 2019.

The effect of functionalization of mesocellular foam silica (MCF) with different organic groups was investigated using stearic acid (SA) as the PCM [66]. Phenyl and methyl groups were chosen as the hydrophobic aromatic and aliphatic moieties, while propyl amine and propionic acid were employed as the basic and acid hydrophilic functional groups. Molten stearic acid was added to the silica matrices in excess. After cooling, the solids were transferred to filter papers and the excess fatty acid was drained out in order to obtain shape-stabilized composite PCMs. Interestingly, a linear correlation between the pore volume and the SA loading was found. All composites exhibit two melting/crystallization processes corresponding to the nanoconfined and interparticle acid phases, as well as a non-melting interface SA layer. A 2.28 ± 0.29 nm layer thickness was computed based on the m.p. decrease of the nanoconfined phase. The volume occupied by the non-melting layer, the nanoconfined crystalline SA and the empty volume were calculated using Equation (6). These volumes were compared with the experimental values computed from the melting enthalpy. The carboxylic acid functionalization reduced the non-melting layer volume by 25% in comparison with the theoretical value, which could be ascribed to supermolecular interactions between the crystalline nanoconfined acid and the functionalized carboxylic groups (Figure 12).

The direct sol-gel method can also be used to prepare functionalized silica composites. For example, the hydrolysis and condensation of vinyltriethoxysilane (VTES) in the presence of PVA as surfactant and methyl laurate (ML) was used to prepare composite PCMs for cold energy storage [145]. While the shape-stability was not reported, composites up to 75% of the heat storage of pristine ML could be prepared. The samples also exhibited increased thermal resistance in comparison with the fatty ester.

The influence of the composites synthesis methods on their thermal properties was studied using lauric acid (LA) and mesocellular foam silica (MCF) [146]. Ethanol solution impregnation was compared with vacuum melt impregnation, melt filtration, grinding, and pressure treatment. The methods based on solid PCM (grinding, pressure treatment) yielded lower nanoconfined phase enthalpy than the solution or melt syntheses. The pressure treatment was also found to destroy the mesocellular foam pore network. The highest heat of fusion and heat storage efficiency value was obtained in the case of vacuum melt filtration, while the highest nanoconfined phase enthalpy and pore fill percentage were obtained for melt filtration. The pore fill percentage is the percent of the total pore volume of the porous matrix occupied by the nanoconfined crystalline phase and non-melting layer. A portion of the pore volume is empty, since the liquid PCM has lower density than the solid nanoconfined phase.

Improving the thermal conductivity of fatty acid-porous silica PCMs through the addition of carbon based materials was also investigated. For example, 1, 3 and 5% wt. carbon nanotubes (CNT) were added to a 32% wt. capric acid–palmitic acid eutectic adsorbed onto fumed silica [147]. The CNT doping did not influence the heat storage properties of the composites (other through decreased fatty acid content), but provided an increase of thermal conductivity up to 0.47 W m^−1^ K^−1^ at 5% wt. CNT versus 0.16 W m^−1^ K^−1^ for the pure CA-PA eutectic. 1, 3 and 5% wt. graphene nanoplatelets (GNP) were also investigated as an additive for palmitic acid-silica NPs composite PCMs [148]. The maximum loading without leakage of the PA-SiO_2_ material was determined to be 70% wt. Thermal conductivity increases with increasing GNP content, from 0.117 to 0.193 W m^−1^ K^−1^ for 0% and 5% GNP, respectively. No significant change in enthalpy was noticed after 100 heating-cooling cycles. A graphene oxide-silica mixed aerogel was synthesized and used for both thermal energy storage and light-to-thermal energy conversion [149]. Octadecanol was melted and impregnated into the aerogel at 75% wt. loading. The composites containing up to 2% GO exhibited lower thermal conductivities than pure octadecanol, but higher than pristine silica aerogels and they could rapidly heat to 50 °C under illumination, proving their light-to-thermal energy conversion. Molten palmitic acid (PA) was impregnated into a hybrid carbon–silica aerogel prepared using 3-aminopropyl triethoxysilane (APTES) as the silica source [150]. The fatty acid–aerogel composite has similar thermal conductivity values as the pure palmitic acid. An 82.2% wt. acid mass fraction was determined by thermogravimetry. Interestingly, the melting point of the composites with both a carbon aerogel and carbon-silica aerogel is decreased in comparison with bulk, while the enthalpy is similar to the value expected based on mass fraction (Table 4). These results suggest that the fatty acid is nanoconfined inside the hybrid aerogel without the presence of a non-melting layer.

Obtaining composite PCMs with lower thermal conductivity is also gaining increased attention as a method for thermal insulation and protection. For example, silica aerogels were prepared and loaded with octadecanol (OD) or PEG 2000 as PCMs [151]. The thermal conductivity of the OD composite was twice as low as that of pure OD, decreasing from 0.25 to 0.12 W m^−1^ K^−1^ after impregnation. Good heat storage efficiencies were obtained for the composites, with 91% and 96% values for the PEG 2000 and OD-loaded samples, respectively.

**Table 4:** Representative porous silica-fatty acids and derivatives nanocomposites.

PCM			Porous Silica Composite				Ref.
Sample	m.p. (°C)	Δ*H_f_* (J g^−1^)	Sample/Synthesis	%PCM (wt.)	m.p. (°C)	Δ*H_f_* (J g^−1^)	
Stearic acid	55.6	176.7	Direct synthesis/TEOS/HCl	60	54.9	109.4	[85]
Dodecanoic acid	44.6	169.0	Gas transport/12.5 nm SBA-15	-	22.3	65.9	[109]
Tetradecanol	36.0	198.0		-	11.4	48.4	
Decanoic acid	30.0	163.0		-	11.1	64.3	
Dodecanol	24.0	196.0		-	0.2	69.5	
Quinary eutectic	12.3	134.4	Melt impregnation/electrospun SiO_2_ fibers	80.2	13.4	107.8	[132]
LA:PA:PAR eutectic	33.1	140.6	Melt impregnation/SiO_2_ NPs + HDPE	75	31.5	104.4	[133]
Lauric acid	44.4	180.8	Direct synthesis	65	42.5	117.2	[124]
Stearic acid	59.9	177.8	Solution impregnation/fumed silica	46	58.8	82.5	[137]
Octadecanol	-	235	Vacuum melt/CH_3_-aerogel	86	-	153.7	[144]
Lauric acid	42.7	166.0	Hexane solution/MCF	83	34.0/ 41.2	123.7	[67]
CA: LA:PA = 61.9:31.0:7.1	15.0	120.2	Melt impregnation/electrospun SiO_2_ fibers	81	13.7	100.9	[131]
Stearic acid	65.2	239.4	Solution impregnation/Tannic acid templated SiO_2_	70	67.1	108.8	[138]
CA:PA: SA = 79.3:14.7:6.0	18.5	139.3	Melt impregnation/SiO_2_ NPs	75	17.2	99.4	[134]
CA:MA = 72:28	21.7	139.2	Direct synthesis	40	21.15	55.6	[126]
Stearic acid	-	221.8	Solution impregnation/MOS	70	-	108.0	[140]
Myristic acid	57.7	184.3	Solution impregnation/WMSN	65	54.7	92.0	[142]
Stearic acid	52.5	172.7	Vacuum melt/SiO_2_ NP	70	52.1	77.6	[122]
Octadecanol	57.2	234.5		70	56.4	47.0	
Lauric acid	42.7	176.1	Vacuum melt/MCF	84	31.5/41.7	128.1	[146]
CA-PA (85:15)	27.5	151.5	Melt/Fumed silica+5% CNT	30.4	25.2	41.2	[147]
Stearic acid	56.6	170.3	Direct synthesis	76	53.8	118.3	[127]
Lauric acid	44.2	165.8	Direct synthesis; TEOS+MTES		42.2	82.7	[129]
Stearic acid	68.4	213.6	Melt impregnation/MCF-COOH	79	58.9/68.8	128.3	[66]
Methyl laurate	4.0	210.1	Direct synthesis, VTES/PVA	71	6.7	151.3	[145]
Palmitic acid	62.8	209.7	Solution impregnation; 5% GNP	70	60.6	128.4	[148]
Octadecanol	57.8	237.8	Melt impregnation/2% GO- SiO_2_ aerogel	75	53	129.6	[149]
1, 8-Cctanediol	62.4	225.1	Direct synthesis, TEOS/HCl	70	61.3	157.7	[128]
Caprylic acid	12.0	139.9	Melt impregnation/silica gel	48	13.8	46.4	[136]
Palmitic acid	50.0	213.1	Melt impregnation/C-SiO_2_ aerogel	82	187.7	43.4	[150]

### 3.3. Polyethylene Glycol (PEG)-Based PCMs

PEG is considered a suitable material for phase change materials, having large heat of fusion, chemical stability and melting points which depend on the degree of polymerization. As in the case of any PCM based on solid-liquid phase transition, leakage is the main disadvantage which could be overcome by incorporation in various matrices. The confinement effect provided by the silica has an important role in the shape stabilization of the materials. PEG-silica composites represent low-temperature heat storage materials, as PEG has a low melting temperature ranging from 3.2 °C to 68.7 °C [152].

Yang et al. studied this confinement effect, by preparing PEG-silica composites through a sol-gel method [153]. Different PEG chains were employed, with M_w_ of 1500, 4000, 6000, and 10,000 Da. The procedure consisted in dissolving different amounts of PEG in ethanol, followed by the addition of TEOS as silica precursor, in an acidic medium. Shape stabilized composites were obtained at 80 °C when the mass fraction of PEG was between 50–80% in the case of PEG 1500. The sample with 80% content of PEG 1500 exhibited the highest content of crystalline phase and the highest heat of fusion among same PEG chain composites, 7.3 J g^−1^, but, much lower in comparison with pure PEG 1500, 148.2 J g^−1^. The explanation of this phenomenon is that the lower amount of PEG, the higher its content embedded in amorphous state in the silica, which prevents it from melting. When the content increases, some of the PEG chains have some part out of the silica framework and can undergo melting. The melting point decreased with increasing the amount of PEG, which was associated to nanoconfinement. The FTIR spectra showed that only physical interactions occurred between PEG and silica. The highest crystallinity and heat of fusion was obtained for PEG 10,000, 48.3% crystalline content and 74.5 J g^−1^, compared to 167 J g^−1^ in the case of free PEG.

Sol-gel synthesis was used to prepare polyethylene glycol M_w_ 4000 (PEG 4000)-silica composites [85]. Poor crystallinity for PEG content below 60% wt. was noticed. With increasing the pore size, an increasing in the enthalpy was observed.

**Figure 13:**
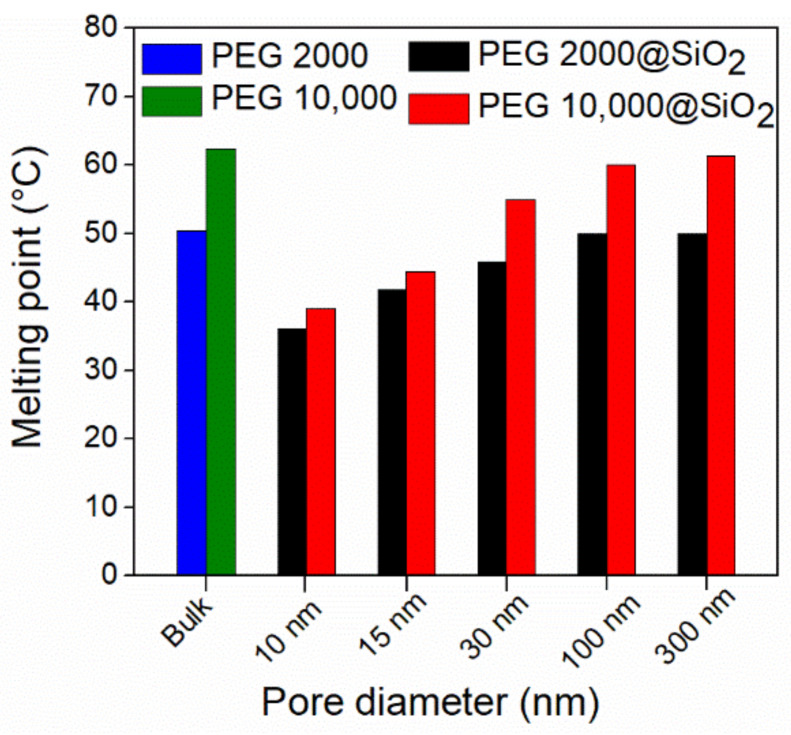
The melting temperature dependence on the pore size of the matrix for PEG 2000 and PEG 10,000 encapsulated into different pore size silica matrix or in bulk. Data from ref. [152].

The relation between the pore size and the melting temperature of PEG was studied, using porous silica with pore sizes between 10–200 nm [152]. The correlation is based on the Gibbs-Thompson equation and the authors compared a simple blending method to a solution impregnation one, in order to see the effect of the nanopores on the melting temperature. Two PEG with molar masses of 2000 and 10,000 were blended with or impregnated into disordered porous silica. The crystallinity of PEG decreases with decreasing the pore size, due to nanoconfinement and irregular arrangement of PEG chains on the silica surface. The melting temperature of PEG shifts to lower values with decreasing pore size, in accordance with the Gibbs-Thompson equation, and the enthalpy of fusion also decreases, as the crystallinity decreases (Figure 13). The supercooling of the composites increases with decreasing pore size, to an extent of almost 6 °C in comparison to the bulk.

A strategy to increase the heat of fusion of PEG/silica composites is to add two types of PEG, with different molecular weights to achieve co-crystallization. Co-crystallization can lead to the increase of the crystalline region because of PEG chains interpenetrations, and therefore an increase in the heat of fusion is obtained. PEG with M_w_ of 2000 and 10,000 Da was used to prepare shape-stabilized composites through sol-gel synthesis, using TEOS as the silica precursor [154]. The optimum ratio between PEG 2000 and PEG 10,000 was found to be 3:1, as this composite had the highest heat of fusion, 108.6 J g^−1^ (Table 5). The DSC analyses showed the presence of two melting peaks, associated to the two types of PEG, overlapping to some extent and confirming the co-crystallization.

Guo et al. also used a sol-gel process for the obtaining of PEG/silica composites, starting with Na_2_SiO_3_ as silica source [155]. No heat of fusion was noticed at 50% wt. PEG due to the nanoconfinement of amorphous PEG in the silica pores. The enthalpy of fusion increased with increasing the PEG content, getting closer to the theoretical value. The formation of a non-melting PEG layer of constant thickness was used to explain the decreased heat storage efficiency.

Carbon fibers (CF) were introduced to a PEG silica composite obtained through the sol-gel method, using 85% wt. PEG and 1–5% CF wt. content in order to increase its thermal conductivity [156]. The enthalpy of fusion and melting temperatures of CF/PEG/SiO_2_ composites were similar to those of PEG/SiO_2_, and lower than that of pure PEG, suggesting that the addition of CF did not exert significant changes on enthalpy or melting temperature. The thermostability of CF containing composites improved when compared to pure PEG. The CF also improved the absorbance of light and its conversion to heat and the thermal conductivities of the composites, proportionally to the CF content. Ca, Mg, and Al metal chloride were used as coagulant in the sol-gel process of obtaining PEG/silica composites, in order to increase the thermal conductivity of the composites without the addition of fillers [157]. The thermal conductivity of the samples increases with the molar weight of PEG and with the addition of metal ions in the synthesis. The metal cations can form coordination bonds with PEG. The best result was obtained for Ca^2+^ ions and PEG 20,000, with a thermal conductivity of 0.41 W m^−1^ K^−1^. The addition of metal ions, however, leads to lower melting and crystallization enthalpies in comparison to those of silica/PEG composites, because PEG chains movement is hindered by the bonds formed with the metal ions. The materials also exhibit high thermal stability. A method of obtaining PEG/SiO_2_ PCMs, starting from silicagel industrial wastes was developed [158]. As previously noticed, PCMs with 50–70% wt. loading exhibited no leakage, while a small leakage in the case of 80% wt. PEG was noticed. The composites have good thermal stability below 350 °C. The enthalpies of the PEG: SiO_2_ PCMs are lower than that of pure PEG, with the highest heat of fusion exhibited by the composite with 80% PEG content, 132.4 J g^−1^ compared to 164.6 J g^−1^ for pure PEG. For the 50% wt. PEG composite an increase in the thermal conductivity from 0.31 W m^−1^ K^−1^ to 0.40 W m^−1^ K^−1^ was noticed.

The presence of a high amount of hydroxyl groups on the surface of silica can affect the thermal capacity of the final composites, because PEG is strongly bound to the surface of the silica through hydrogen bonds, which hinders the crystallization process. Serrano and coworkers studied these interactions by controlling the condensation process, through a second catalysis step, using different quantities of NaOH [159]. The authors optimized the process for a short gelation time and complete hydrolysis of TEOS, however, the FTIR spectra and DSC analysis confirmed the presence of hydrogen bound PEG. The melting of the PCMs took place at higher temperature than the crystallization process, indicating supercooling. The supercooling was larger for the composites than for pure PEG probably due to the interactions between silica and PEG. The lowest content of non-melting PEG was obtained in the case of the material neutralized with a slight excess of NaOH, because the large content of silanol groups on the silica surface diminish the amount of PEG bound to the OH groups. This composite presented an enthalpy of 113.8 J g^−1^, in comparison with 146.7 J g^−1^ for pure PEG.

A combined sol-gel method with acrylic acid (AA) in situ polymerization was used for obtaining PEG/SiO_2_/AA composites with PEG as PCM and cross-linked silica-AA network as support [160]. The advantage of this method is that the final material can be molded into any shape, having good shape, thermal stability and thermal reliability. The highest value for the enthalpy of fusion was obtained for the composite with the highest mass fraction of PEG (44.3%), 91.9 J g^−1^ versus 171 J g^−1^ for free PEG (Table 5). The measured heat of fusion values are higher than the theoretical ones, which is atypical to these types of composites as they are usually restricted by the nanoconfinement effect. This could be explained by the rearrangement and association of the PEG chains as the crosslinking reaction occurred. The effect of polyacrylic acid on the SiO_2_/PEG composites, for different PEG chains was also studied and no increase in enthalpy when compared to the theoretical value was found [161].

PEG/SiO_2_ can be used to control porous asphalt concrete temperature, which can suffer deformation due to high temperatures during summer [162]. PEG-4000/SiO_2_ composites were prepared through a sol-gel process, with the 70% wt. PEG composite having the highest heat of fusion (100 J g^−1^). The composite was added into porous asphalt instead of fine aggregates and could reduce the internal temperature of asphalt concrete. A PEG/SiO_2_/dye composite was studied for light-to-thermal energy capture, and the results showed that the dye containing composites reduced the degree of supercooling of PEG, and exhibited a melting enthalpy of 167 J g^−1^ for 88.5% wt. of PEG in comparison to 212.8 J g^−1^ for pure PEG [163]. This composite showed good light-to-thermal conversion and thermal reliability after 300 phase transition cycles. Wood was impregnated with silica and PEG to obtain a shape stabilized phase change material that can be used as a building material [164]. The wood was loaded either with only PEG or with PEG/SiO_2_. PEG/SiO_2_ treated composites exhibited lower enthalpies, but improved the thermal stability, shape stability, and reliability.

Other methods besides the direct sol-gel synthesis can be used for the obtaining silica-PEG composites. PEG impregnations from solution or through vacuum melting have been employed [165,166]. Vacuum melting leads to lower heat storage efficiencies due to a higher content of PEG molecules being present in the non-melting layer. In comparison with the sol-gel method, molten impregnation leads to high enthalpy values, being among the highest values obtained in other studies (Table 5). An impregnation method was also used to prepare composite PCMs [167], starting from PEG 4000, and a nanoflower-like silica structure. 136.6 J g^−1^ heat of fusion values and no leakage were found at 80% wt. PEG. The composite had a melting temperature of 50.8 °C and a crystallization temperature of 41 °C, with the presence of the silica matrix reducing the degree of supercooling.

PEG crystallization behavior can be influenced by different functionalities attached to the silica surface. Dopamine was bound to the silica surface through hydrogen bonding [168]. PEG (M_w_ 4000) was then loaded through vacuum impregnation from an ethanolic solution. The polydopamine coating led to the formation of a more crystalline PEG phase, as the heat of fusion of polydopamine composites is higher than the enthalpies of the PEG-silica composites: 73.8 J g^−1^ versus 67.2 J g^−1^ for 70% wt. PEG. This can be explained by the reduction of PEG-silica surface interaction due to the new hydrogen bonds formed between polydopamine and silica surface, leaving only some imino groups to interact with PEG. The same strategy was adopted for SBA-15 type silica [169] and the results showed no leaking in the case of the 70% wt. PEG 2000 composite, with good thermal stability. Two endothermic and exothermic peaks were observed in the case of polydopamine composites, which could indicate the presence of a nanoconfined and bulk phase.

**Figure 14:**
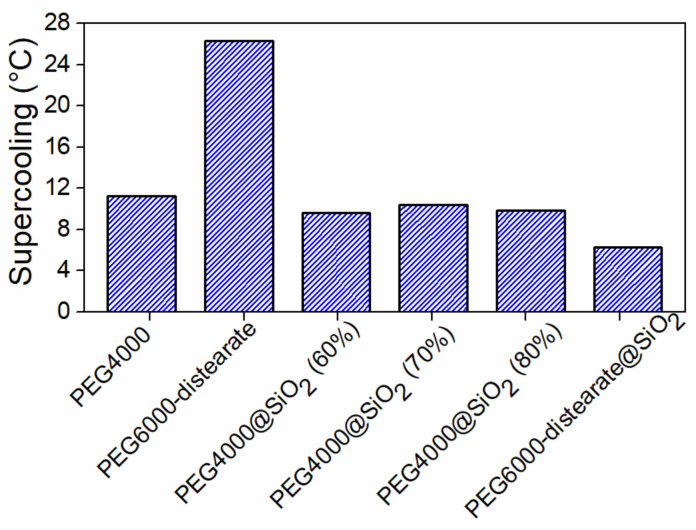
The degree of supercooling for PEG 4000, PEG 6000-distearate, PEG-SiO2 with different PEG weight fractions and PEG-distearate-SiO_2_. Data from ref. [167,170].

Fatty acid esters of PEG can be used as phase change materials with higher melting points than pure PEG [170]. 61.6% wt. PEG 6000 distearate was encapsulated into a silica shell through the sol-gel method. The sample showed two phase transitions with temperatures 20 °C and a 52.9 °C and a melting enthalpy of 69.7 J g^−1^. The supercooling effect was minimized by the esterification of the end groups of PEG (Figure 14).

PCMs were also used as additives in cement mortar for buildings external walls in order to reduce the indoor temperature fluctuations [170,171]. PEG 600 was impregnated into fumed silica under vacuum. A maximum PEG: silica ratio of 1.6:1 could be loaded with no leakage. These composites were then used in mortar cement mixtures as plaster for several wall specimens, in order to test their properties. The experimental 71.6 J g^−1^ heat of fusion value is close to the 72.8 J g^−1^ theoretical enthalpy. The presence of the PCM, however, reduces the compressive strength of the mortars, and delays the cement hydration processes.

Li et al. reported obtaining PEG/SiO_2_ composites, inspired by mesoporous silica synthesis, in which PEG 6000 is completely encapsulated into a silica shell [172]. The melting enthalpy of the composite is 164.9 J g^−1^, close to that of pure PEG, 178.6 J g^−1^ and it corresponds to a mass fraction of 97.3%, calculated from TGA measurements. MCM-41 type mesoporous silica was also used for obtaining shape-stabilized PEG 2000 phase change materials [173], with PEG loaded through solution impregnation. It was noticed that PEG did not undergo melting even at 70% wt. when impregnated in MCM-41 silica. However, the phase change was noticed starting from 30% wt. when the surface was modified with NH_2_ groups. The melting enthalpy of 60% wt. PEG-MCM-41-NH_2_ silica composite was 58.8 J g^−1^, lower than the theoretical value.

**Table 5:** Representative examples of different types of PEG as phase change materials in porous silica composite PCMs.

PCM			Porous Silica Composite				Ref.
Sample	m.p. (°C)	Δ*H_f_* (J g^−1^)	Synthesis	%PCM (wt.)	m.p. (°C)	Δ*H_f_* (J g^−1^)	
PEG 600	18.5	118.2	solution impregnation	62	19.6	71.6	[171]
PEG 2000	52.5	153.0	vacuum impregnation	60	50.8	58.76	[173]
PEG 4000	53.8	202.1	vacuum impregnation	80	50.8	136.6	[167]
PEG 4000	59.1	183.4	vacuum impregnation	70	57.8	121.7	[166]
PEG 4000	54.5	192.4	vacuum impregnation	70	53.0	73.8	[168]
PEG 6000	61.7	178.6	sol-gel	97.3	60.4	164.9	[172]
PEG 6000 distearate	52.9	145.1	sol-gel	61.6	52.9	69.7	[170]
PEG 6000	59	171	sol-gel	44.3	56.8	91.9	[160]
PEG 6000	61.4	212.8	sol-gel	88.5	58.4	167.0	[163]
PEG 1000	35.1	146.7	sol-gel	84.5	35.2	113.8	[159]
PEG 1500	41.1	164.6	sol-gel	38.4	80.0	132.4	[158]
PEG 2000 + PEG 10,000	51.7/62.4	180.6/ 170.9	sol-gel co-crystallization	36	56.5	108.6	[165]

The PEG-silica composites show good properties, such as high enthalpy of fusion, thermal stability and reliability and no leakage up to 80% wt., which make them suitable as thermal energy storage materials. They have some drawbacks, such as the large extent of supercooling and strong interaction with the silica surface, but these can be minimized through either PEG chain modification or functionalization of the silica surface.

### 3.4. Small Organic Compounds

Various organic compounds with small molecules can be used to reversibly store thermal energy through the solid–liquid phase transition. High heat of fusion values are obtained for compounds possessing multiple functional groups capable of forming hydrogen bonding with each other. An example in this case is acetamide, which has high capacity for forming hydrogen bonding and small molar mass and it is therefore a suitable PCM for adsorption into porous silica [174]. The class of small organic compounds often has large heat of fusion values, but suffers from supercooling.

*D*-Manitol was incorporated into silica microparticles through direct sol-gel synthesis at a pH of 3.0 [175]. The composite exhibited reliability after 100 heating-cooling cycles and a lower degree of supercooling than the pure sugar compound. The supercooling was reduced from 44.2 to 11 °C after sol-gel silica encapsulation, while the thermal conductivity increased from 1.32 to 1.77 W m^−1^ K^−1^.

An aerogel obtained through the supercritical drying of methanol was impregnated with erythritol, up to a maximum loading of 85% wt. [176]. The aerogel had an average pore diameter of 53 nm and a total pore volume of 4.39 cm^3^ g^−1^. The resulting composite had a high heat storage capacity of ~290 J g^−1^.

Hard-shell silica microcapsules (HSMCs) with a diameter of 10–15 μm were obtained by the double-emulsion method and used to encapsulate trimethylolethane hydrate [177]. The latent heat and transition temperature of the hydrate can be tailored by changing its concentration. Thus, 45% wt. PCM could be loaded into the HSMCs with no apparent loss in heat storage capacity.

The solid–solid phase transition of *tris* (hydroxymethyl) aminomethane was studied under nanoconfinement in silica gels and porous glasses with pores ranging from 6 to 200 nm [178]. Both the transition temperature and heat of fusion decrease when lowering the pore diameters. The pure *tris* (hydroxymethyl) aminomethane exhibits large supercooling, which is reduced when the pore size is lower than 30 nm.

### 3.5. Hydrated Salts

The use of hydrated salts as phase change materials is based on their low cost and high heat of fusion. The most known example is the sodium acetate trihydrate, which is found in commercial products. Hydrated salts have large melting enthalpy values, often in excess of 200 J g^−1^ with melting points between 0–100 °C (Table 6). However they also suffer from supercooling and incongruent crystallization, which leads to phase separation between the crystallization water and the salts during heating-cooling cycles. Thus porous silica matrices are also studied with the goal of decreasing supercooling and phase separation.

The addition of silica nanoparticles with diameters between 12 and 50 nm on the thermal properties of Na_2_SO_4_·10H_2_O, Na_2_HPO_4_·12H_2_O, and Na_2_S_2_O_3_·5H_2_O was investigated through thermal history and calorimetry [179]. The addition of up to 7% nanoparticles reduced the supercooling of the hydrated salts crystallization. The MgCl_2_·6H_2_O-Mg(NO_3_)_2_·6H_2_O system was studied for thermal energy storage [180]. Compositions between 35 and 50% wt. MgCl_2_·6H_2_O produced only one endothermic melting peak. 5–20% wt. Fumed silica was then added to the salt mixture, obtaining shape stabilized composites at 15% or higher fractions. The salt mixture containing 41.3% MgCl_2_ proved to be unstable in a 100 heating-cooling cycle test, as the water separates from the salts. However, the fumed silica composite shows only a 5% loss of enthalpy after 100 cycles, indicating much better reliability than the hydrated salt mixture. CaCl_2_·6H_2_O doped with 2% SrCl_2_ as a nucleating agent was impregnated into three types of commercial silica nanoparticles [181]. The maximum salt hydrate loading varied between 70% and 75% wt. depending on the SiO_2_ NP size. The heat of fusion of a 75% hydrated salt composite decreased from 148.2 J g^−1^ to 138.0 J g^−1^ after 500 thermal cycles, indicating high reliability.

Not only inorganic hydrated salts can be used for thermal energy storage. Lan et al. have used *n*-alkyl zinc chloride complexes having the formula (*n*-C_n_H_2n+1_NH_3_)_2_ZnCl_4_ as PCMs [182,183]. Silica gels with pore sizes varying between 15 and 200 nm were used as matrices for the C_14_ complex [183]. The solid-solid transition of the complex shows the same dependence on pore size as solid-liquid transitions. Both the transition temperature and heat of fusion are decreased with an amount proportional to 1/*d*, similar to the Gibbs-Thompson equation.

One of the biggest drawbacks of hydrated salts is their large supercooling and phase separation between the salts and water during use. A comprehensive study using sodium acetate trihydrate as the PCM, carboxymethyl cellulose (CMC) and silica gel as matrices, and Ag nanoparticles as nucleating sites found that the supercooling can be reduced and the heat storage properties can be increased for the optimum composition. This consisted of 85% CMC in the CMC + SiO_2_ matrix and 0.5–0.7% wt. Ag NPs [184]. A silica gel was prepared by the sol-gel method and coated with 10% PVP [185]. Na_2_SO_4_·10H_2_O-Na_2_HPO_4_·12H_2_O mixture was used as the PCM at 70% wt. loading. The coated matrix exhibited higher stability during cycling, as well as m.p. and heat of fusion decrease in comparison with the hydrated salt fraction, indicating that the stability is caused by the PCM nanoconfinement in the silica matrix. A follow-up study showed that increasing the silica pore size and decreasing the silanol group density yields the highest enthalpy at 70% wt. hydrated salt loading [186].

The addition of 0.5–4% wt. fumed silica to Na_2_HPO_4_·12H_2_O was studied with the aim of reducing supercooling [187]. The smallest supercooling degree was found for the 0.5% wt. fumed silica sample, while the thermal conductivity increases with increasing matrix ratio. Hydrophilic fumed silica was added to tetra-*n*–butyl ammonium bromide (TBAB) solutions (36–44% wt.) in order to obtain form stable PCMs [188]. 1.5–3.5% wt. Na_2_HPO_4_·12H_2_O was added as a nucleating agent, while the fumed silica ratio was varied between 20–35% wt. The addition of 2.5% Na_2_HPO_4_·12H_2_O yielded composites with the lowest supercooling degree while 30% fumed silica was sufficient to obtain form stable materials with no leakage. The total heat storage capacity of the composite decreased from 134.0 to 111.6 J g^−1^ after 100 heating-cooling cycles. A similar amount of fumed silica (30%) was also needed to obtain shape-stabilized PCMs using the Na_2_SO_4_·10H_2_O-Na_2_HPO_4_·12H_2_O eutectic and Na_2_SiO_3_·9H_2_O as nucleating agent [189].

A non-eutectic mixture of urea and sodium acetate trihydrate was melted and mixed with fumed silica as the matrix, 1.5% wt. Na_2_HPO_4_·12H_2_O as nucleating agent and 2% wt. sucrose as thickener [190]. 30% wt. silica was sufficient to prevent leakage and decreased supercooling to 1.1 °C. In addition, the sample had good reliability after 200 heating-cooling cycles, with a loss of only 2% of its enthalpy.

The decrease of the available pore volume caused by the presence of the non-melting layer was used to quantify the nanoconfined enthalpy of hydrated salts PCMs adsorbed into SBA-15 [191]. The non-melting layer was approximated using the fractal dimension model (see Section 2.4).

**Table 6:** Representative examples of hydrated salts as phase change materials in porous silica composite PCMs.

PCM			Porous Silica Composite				Ref.
Sample	m.p. (°C)	Δ*H_f_* (J g^−1^)	Synthesis	%PCM (wt.)	m.p. (°C)	Δ*H_f_* (J g^−1^)	
Na_2_SO_4_·10H_2_O-Na_2_HPO_4_·12H_2_O	36.7	226.9	Melt impregnation/sol-gel SiO_2_ + PVP	70	30.1	106.2	[185]
			Melt impregnation/sol-gel SiO_2_	70	28.5	67.5	[186]
MgCl_2_.6H_2_O:Mg(NO_3_)_2_·6H_2_O (41.3:58.7)	58.8	118.5	Melt impregnation/fumed silica	85	54.3	88.1	[180]
Na_2_SO_4_·10H_2_O-Na_2_HPO_4_·12H_2_O	-	221.4	-	70	-	64.1	[191]
TBAB:H_2_O (4:6)	11.8	211.9	Melt impregnation/hydrophilic fumed silica	70	8.3	134.0	[188]
CaCl_2_·6H_2_O	29.4	199.9	Melt impregnation/SiO_2_ NPs	75	25.1	148.2	[181]

### 3.6. Molten Salts

Molten salts are typically employed for high temperature applications as PCMs. Most molten salts are corrosive and can react with the silica matrix at elevated temperatures, thus a careful selection of the salt nature, silica matrix and operating temperature must be carried out.

A 1:1 wt. sample consisting of sodium sulfate and silica was prepared by sol-gel synthesis starting from sodium silicate [192]. The pore diameters are around 20 nm in the silica framework. The composite exhibits gradual loss of enthalpy upon repeated heating and cooling, with 15% of its heat storage capacity lost after 100 cycles. The direct sol-gel method was also used to prepare a 60% wt. NaNO_3_/SiO_2_ composite starting from sodium silicate [193]. The composite maintains its shape at elevated temperatures and it has thermal stability up to 500 °C. Na_2_SO_4_/SiO_2_ particles were embedded into SiO_2_-Al_2_O_3_ aerogels up to 61% wt. loading [194]. The unloaded aerogel had a pore volume of 4.28 cm^3^ g^−1^ while the PCM sample still retained 0.57 cm^3^ g^−1^ total pore volume even after treatment at 1000 °C. A total enthalpy of 125 J g^−1^ with a melting point of 871.2 °C was obtained for the composite containing 61% wt. Na_2_SO_4_/SiO_2_ particles.

The sol-gel method was also employed to obtain Li salts-silica composites with salt loading up to 60% wt. [195]. While Li_2_CO_3_ and CH_3_COOLi·H_2_O did not yield shape-stabilized PCMs due to reactions with the silica framework, LiCl and LiNO_3_ could be used for heat storage applications. In particular, the LiNO_3_-SiO_2_ composite prepared using 60% wt. salt retained 236.3 J g^−1^ after 50 heating-cooling cycles.

The addition of 10% wt. SiO_2_ or SiC nanoparticles to a mixture of Na_2_CO_3_-K_2_CO_3_ eutectic containing 45% wt. MgO was investigated [196]. The sintered samples containing the SiO_2_ nanoparticles exhibited similar m.p. and thermal conductivity values as the starting material, while the heat of fusion was reduced due to the lower eutectic mass fraction (Table 7).

KCC-1 mesoporous silica with radially ordered pores which increase in diameter from the center of the particles to the exterior has been used to create PEG, LiNO_3_ and Na_2_SO_4_ composite PCMs [166]. 70% wt. loadings resulted in shape-stabilized materials, which exhibited little heat loss after 20 heating–cooling cycles and high heat storage potential (Table 7).

A study of NaCl-CaCl_2_ eutectic impregnation into mesoporous silica with 6.3 and 8.1 nm mesopore diameters was performed by our group [197]. The initial eutectic salt fraction was varied from 70–95% wt. in the final composite. No melting/crystallization processes were noticed for salt loadings lower than 90% wt. This phenomenon arises from the reaction of calcium chloride with the silica matrix, yielding calcium silicate species. An initial NaCl-CaCl_2_ loading higher than 80% wt. is required for the complete formation of the calcium silicate matrix. The resulting composites have specific heat capacity of 1.0–1.1 J g^−1^ K^−1^, thermal stability up to 700 °C, and shape-stability above their melting point, indicating that the mesoporous silica can be used as a reactive matrix for obtaining shape-stabilized PCMs containing molten salts.

**Figure 15:**
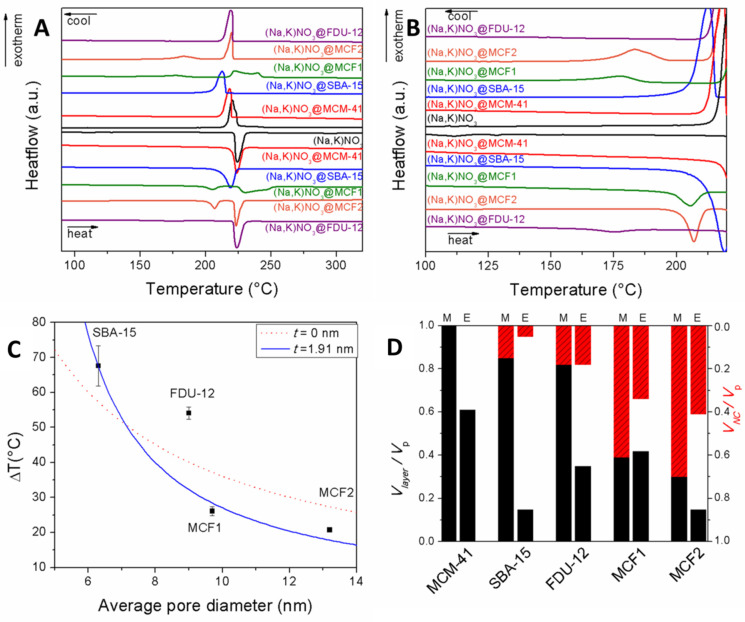
(**A**,**B**) DSC analyses of NaNO_3_-KNO_3_/MSN composites, (**C**) determination of non-melting layer thickness from Equations (2) and (5) and (**D**) comparison of pore volume fraction occupied by the non-melting layer and nanoconfined phases using a theoretical model. M represent model and E experimental data. Adapted with permission from ref. [74] Copyright Elsevier B.V. 2020.

A 1:1 mol. molten NaNO_3_-KNO_3_ eutectic was adsorbed into five types of mesoporous silica nanoparticles at 90% wt. [74]. The MSN matrices consisted of hexagonal 2.7 nm pore diameter MCM-41 and 6.3 nm diameter SBA-15, as well as FDU-12 with a cubic arrangement of “ink-bottle” 3.7/9.0 nm diameter mesopores and two types of mesocellular foam silica (MCF) with 9.7/22.4 nm and 13.2/29.8 nm diameter disordered pores. With the exception of MCM-41, all other MSN yielded shape-stabilized materials. Interestingly, two melting and two crystallization processes were evidenced, corresponding to the mesopore nanoconfined and interparticle salt phases (Figure 15A,B). The heat of fusion decrease with respect to the theoretical value based on the eutectic mass fraction indicates the presence of a non-melting interface layer. Using the m.p. depression and Gibbs–Thompson equation, the thickness of the non-melting layer was computed at 1.9 nm (Figure 15C). Using the model presented in Figure 5, the pore volume fractions occupied by the non-melting layer, the crystalline nanoconfined salt phase and the empty pore volumes were calculated and compared with the results determined from experimental measurements (Figure 15D). These results show a direct increase in nanoconfined volume and decrease of the non-melting layer with increasing pore diameter, as well as a corresponding reduction of the empty pore volume, suggesting that mesoporous silica with larger pore diameters are the best matrices for molten salt PCMs.

**Figure 16:**
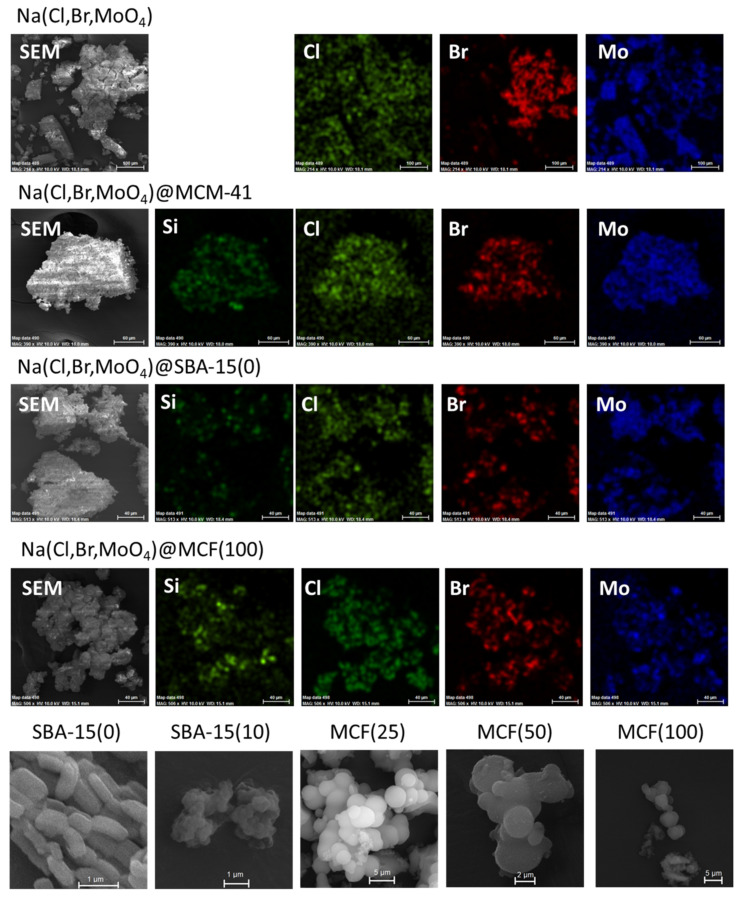
Scanning electron microscopy of silica matrices, ternary NaCl-NaBr-Na_2_MoO_4_ molten salt and composites PCMs, as well as elemental distribution maps. Reproduced with permission from ref. [198]. Copyright Elsevier B.V. 2020.

Melt impregnation was also used to load a ternary NaCl-NaBr-Na_2_MoO_4_ salt eutectic into various mesoporous silica matrices at 80% wt. mass fractions [198]. This molten salt eutectic exhibits both a solid-liquid transition at 522 °C and a solid-solid phase change at 454 °C which could be used for high temperature heat storage. High temperature optical microscopy confirmed that all composites retain their shape at temperatures higher than their melting points. The m.p. of each composite is decreased with 5–10 °C with respect to the pure eutectic, signifying nanoconfinement effects in the interparticle space. Similar decreases are noticed for the solid-solid transition as well. Using the Gibbs-Thompson equation, the diameter of the interparticle salt phase was computed at 60–150 nm, higher than the 2.7–29.8 nm pore size. All composite samples have high thermal stability up to 700 °C. The sample obtained using MCM-41 mesoporous silica matrix with the smallest pore diameter (2.7 nm) exhibited high total heat of fusion (168.9 ± 8.7 J g^−1^) which remain unchanged after 50 heating-cooling cycles. The morphology of the mesoporous silica matrices varies from agglomerated rods for SBA-15 to spheres for mesocellular type silica (Figure 16). The samples containing the ternary molten salt mixture were shown to have an even distribution of the salt species and the silica matrices using electron microscopy analyses (Figure 16).

A comparative study between direct sol-gel synthesis and melt impregnation has been carried out using Na_2_SO_4_·10H_2_O as the starting salt [199]. The sodium sulfate fraction was varied between 20% and 60% wt. in the sol-gel syntheses. The heat of fusion values agree with the initial salt ratio. Vacuum melt impregnation into silica gels yield similar enthalpies and melting points, suggesting that the silica surface silanol groups do not influence the heat storage process.

**Table 7:** Representative molten salt-porous silica composites for thermal energy storage.

PCM			Porous Silica Composite				Ref.
Sample	m.p. (°C)	Δ*H_f_* (J g^−1^)	Synthesis	%PCM (wt.)	m.p. (°C)	Δ*H_f_* (J g^−1^)	
Na_2_SO_4_	888.7	167.1	Direct sol-gel synthesis	50	886.0	82.3	[192]
NaNO3	308.0	189.0	Direct sol-gel synthesis	60	302.0	108.0	[193]
LiNO3	253.8	369.9	Melt impregnation/KCC-1	70	251.3	292.2	[166]
Na_2_CO_3_-K_2_CO_3_/MgO	702.9	81.4	Sintering/SiO_2_ NPs	90	703.6	76.2	[196]
NaCl-CaCl_2_ (1:1 mol)	499.5	208.2	Reactive melting/8.1 nm MSN	95	499.1	60.8	[197]
Na_2_SO_4_	886.7	167.1	Direct sol-gel synthesis	60	886.9	100.8	[199]
LiNO_3_	-	-	Direct sol-gel synthesis	60	232.8	236.3	[195]
NaNO_3_:KNO_3_ (1:1 mol)	221.8	96.9	Melt impregnation/MCF	90	201.0 221.2	78.7	[74]
Na(Cl, Br, MoO_4_)	454.5 522.1	78.9 137.3	Melt impregnation/MCM-41	80	450.4 514.4	52.8 111.2	[198]

### 3.7. Metals, Alloys and Elemental PCMs

Metals and alloys can be used as PCMs. While most metals have low gravimetric heat of fusion, their high density makes them attractive for applications requiring high volumetric heat storage. There are only a few reports to date on the synthesis and characterization of metal-porous silica composites.

A mixture of Ge and Sn embedded into a silica matrix was found to exhibit different melting and crystallization behavior, depending on heating and cooling rate [200]. Under fast temperature change rates the nanocrystals formed an alloy, while slow rates resulted in a more thermodynamically favored state of separated metal phases. Indium nanoparticles could be encapsulated into sol-gel silica, at a In: TEOS = 1:10 wt. ratio and used for thermal management of heterogeneous reactions [84].

Wei et al. proposed a novel composite alloy of Al and Si encapsulated into an Al_2_O_3_ matrix with tunable enthalpy and melting range [201]. The composites are prepared starting from Al powders which react with TEOS in the presence of surfactants. The silica shell formed on the Al particles is reduced to Si during the heat treatment and forms alloys with variable composition depending on the initial conditions. In this way, composites with a melting range of 574.0–641.4 °C and heat of fusion values of 248.6–331.0 J g^−1^ could be obtained.

## 4. Conclusions

Thermal energy storage through phase change materials is attracting increased interest in recent years as a cost-effective energy storage solution. Nevertheless, most bulk substances able to act as phase change materials exhibit a range of undesirable properties, such as supercooling, phase separation, volume expansion during phase transition, leakage, corrosion and decreased reliability during multiple heating-cooling cycles. One of the most promising solutions for addressing these drawbacks is obtaining composite materials using porous matrices, which confine the liquid PCM while retaining their macroscopic shape. Porous silica nanomaterials offer high thermal and chemical stability, large and adjustable pore sizes and volumes and ability to adsorb more phase change materials than their own weight. The most used porous silica matrices include silica gels, nanoparticles, mesoporous silica, aerogels, and xerogels. The confinement of the heat storage compounds into the nanometer scale silica pores accommodates the volume change during phase transition, increases the interactions between the molten PCMs and the matrix surface and limits their contact with the external medium, alleviating most of their drawbacks. Because silica nanomaterials have low conductivity, most composite PCMs are also good thermal insulators. Various solutions could be applied if increased thermal conductivity is desired; most of them are based on the addition of carbon materials, such as graphene oxide or expanded graphite.

The nanoconfinement of phase change materials into porous silica also gives rise to specific effects. The melting point of solid crystalline phases is decreased with respect to bulk and this effect can be larger than 10 °C for mesopores. Porous matrices with smaller mesopores in the 2–40 nm diameter range and high specific surface area, such as aerogels and mesoporous silica, exhibit a non-melting interface layer between the silica pore surface and the confined phase change materials. This layer does not participate in the phase transition, lowering the overall heat storage efficiency. Maximizing the pore volume to surface area ratio and pore diameter ensures that the volume occupied by the non-melting layer is minimized, thereby increasing the heat storage capacity and efficiency. Since the non-melting layer is similar to the liquid phase, the thermal conductivity of these composites is usually increased with respect to bulk PCMs below their melting points.

The thickness of the non-melting layer can be experimentally determined from the melting point depression of the nanoconfined phase, if the pore diameters are known and have narrow size distribution. The volume occupied by the non-melting layer can also be approximated. One simple calculation for this volume is simply the ratio of usable pore diameter raised to a power equal to the fractal dimension of the matrix. More complex determinations are based on geometric considerations of the pore shape or volume, determined from porosimetry measurements. Both methods allow for the determination of theoretical enthalpy lost due to the non-melting layer and the enthalpy of the nanoconfined phase. In practice, a part of the pore volume remains empty due to the difference between the molar volume of the solid and liquid PCM. The empty pore volume can accommodate the volume change during phase transition and it is an integral part of obtaining shape-stabilized materials.

Paraffins, poly ethylene glycols, fatty acids and alcohols make up the majority of PCMs used together with porous silica matrices. Salt hydrates, molten salts, metals and organic compounds with small molecular mass and high capacity for hydrogen bonding have also been investigated as the active heat storage phases. The composites are usually obtained either through direct sol-gel synthesis of the silica framework in the presence of the PCM species or through the impregnation of molten PCMs or their solutions into porous silica matrices. The direct sol-gel synthesis is a simpler method, but the obtained composites are typically limited to 60–70% wt. PCM loading. In contrast, impregnation methods can make use of porous silica with higher pore volumes and usually yield at least 70% impregnated PCM loading while maintain shape stability. Composite materials with heat of fusion values in the 100–200 J g^−1^ range can be obtained. These materials often have good reliability, with negligible change in enthalpy after 100 heating–cooling cycles. The thermal conductivity of the composites can be tailored between 0.1–2.0 W m^−1^ K^−1^ by introducing additional thermally conductive components such as carbon nanomaterials at mass fractions up to 10% wt.

The existence of a non-melting layer for most composites containing porous silica nanomaterials is more pronounced at low PCM loadings. Most materials exhibit negligible heat of fusion under 50% wt. loadings. The maximum PCM loading fraction while maintaining shape stability is influenced by both the nature of the PCM and of the porous silica matrix. In general, the highest mass fractions can be obtained by PEG, followed by fatty acids, while paraffins exhibit the lowest loadings for organic PCMs. This is true for pristine silica matrices containing silanol groups on the surface. The percentage of the active heat storage compound present as a non-melting layer follows the same trend. Typically, materials with 60% wt. PEG have most of the organic PCM as a non-melting layer, while lower loading levels of 50% wt. result in the same behavior in the case of fatty acids. Functionalization with hydrophobic organic substituents reverses the trend: higher mass loadings are obtained for paraffins and lower for PEG, with the same variation in the volume occupied by the non-melting layer. These variations show the importance of the supermolecular bonds between the silica surface and the incorporated PCMs. High attractive interactions, such as those between silanols and PEG or alkyl groups and paraffins, lead to a more complete filling of the pores and of the non-melting layer. This effect can be noticed even for inorganic PCMs, for example hydrated salts interacting through hydrogen bonding with the silanol groups. Tailoring the surface of the silica matrix can be used to decrease the volume occupied by the non-melting layer and therefore increase heat storage capacity. Amino and carboxylic acid groups were shown to have this effect for PEG and fatty acids, respectively.

The effect of the silica matrix on the PCM loadings and thus on the total heat storage capacity can be understood from the basis of available pore volume. In general, matrices with lower pore volume such as silica nanoparticles and fused silica exhibit the lowest PCM loading while maintaining shape-stability. Typical values are at most 70% wt. Intermediate loading values up to 75% wt. are obtained in the case of direct sol-gel synthesis of the composite PCMs, while the highest values, often in excess of 80% wt. can be obtained for high porosity matrices such as aerogels and mesoporous silica. Since mesoporous silica can be obtained as nanoparticles or microparticles, these materials will often exhibit two separate melting and crystallization effects, corresponding to the PCM phase encapsulated into the mesopores and into the interparticle phase. Shape-stability occurs through capillary action in the interparticle space, similarly as for materials containing nanoparticle matrices. Aerogel-based samples offer the possibility of obtaining monoliths, so this effect is less frequently encountered. The volume of the non-melting layer and therefore the lost heat of fusion roughly follows the same trend as the maximum PCM loading. An important distinction is that since the non-melting layer is an interface layer, its volume is actually proportional to the matrix specific surface. Strategies for improving the heat storage capacity of PCMs containing porous silica nanomaterials therefore should maximize the available pore volume while decreasing the surface area, in effect increasing the pore size. Several reports have experimentally shown that major loss of heat storage occurs when the pore diameter decreases below 30–40 nm.

The PCM impregnation method also influences the thermal properties of the resulting materials. Generally, the best results in terms of PCM loading and total heat storage are obtained through impregnation of melted PCMs, especially under vacuum. Solution syntheses offer lower loading, but in general still higher than the direct sol-gel method. Other properties beside total heat storage capacity are also affected by the silica matrices. Thermal reliability, the capacity of the sample to retain its heat storage under repeated heating-cooling cycles, is often improved after impregnation into porous silica nanomaterials. This effect is most pronounced in the case of hydrated salts, which are prone to phase separation of the hydration water during use. Supercooling is another property which is influenced by the porous matrix. Supercooling often occurs in the case of PEG or hydrated salts. Nanoconfined PCM phases most often exhibit supercooling, as a consequence of the different crystallization and melting behavior under nanoconfinement. While melting starts from the interface with the silica pores, crystallization starts from the volume and thus is kinetically hindered. Depending on the silica pore size, the crystallization of the interparticle phase can continue inside the pore volume, decreasing supercooling. The addition of nucleating agents at low weight fractions or silica surface functionalization can be employed to reduce the degree of supercooling. Thermal and chemical stability are often similar of better for the silica-based PCMs in comparison with the pristine PCMs. There are however two exceptions to this rule. First, capillary evaporation for substances wetting the pore walls is favored, as quantified by the Kelvin equation. Coupled with the increase in surface area caused by nanoconfinement, PCMs with high vapor pressures are therefore more susceptible to evaporation. The second exception is the reactivity of some materials towards silica at high temperature. Examples include non-alkali molten salts which can form silicates, molten salts with high basicity such as carbonates or hydroxides and molten metals more electropositive than silicon. Overall, the incorporation of phase change materials into porous silica nanomaterials leads to the improvement of several key properties relating to the long-term storage of thermal energy, as well as offering the possibility of tailoring the melting points, heat storage capacity, thermal reliability, and conductivity of the samples.

## 5. Outlook

Significant progress has been made in obtaining and characterizing composite PCMs containing porous silica matrices which can operate near room temperature, in the 15–80 °C range. These materials can be used in various applications such as passive building heating and cooling, electronics protection, safety equipment, food protection, waste heat capture and reutilization. In contrast, applications requiring high temperatures above 200 °C or cold storage below 0 °C have received less attention. One of the key features of porous silica matrices is their versatility in tailoring their surface properties through functionalization with organic groups or doping of the framework. This key property has received limited attention beyond matching the hydrophobic character of some phase change materials with that of the silica surface. The field of shape-stabilized phase change materials based on porous silica matrices is expanding, but most of the studies are still at laboratory scale. Only several reports have addressed practical aspects of product development and created prototypes.

This review highlights the progress in the area of phase change materials obtained using porous silica matrices. While this is a relatively new area of study, the interest towards it has been increasing in recent years. This interest is explained by the fact that creating composite phase change materials can alleviate most drawbacks associated with the use of pure PCMs, while retaining good heat storage capacity, efficiency, and reliability. Furthermore, there is a large variety of both potential heat storage compounds and porous silica matrices, ensuring that this class of materials can address multiple applications.

## Figures and Tables

**Table 1:** Comparison of the different heat storage strategies.

	Heat Storage	Sensible Heat	Latent Heat	Chemical Heat
Properties				
Energy density		<600–800 kJ kg^−1^ 0.8–1.7 J g^−1^ K^−1^ [4]	~100–1800 kJ kg^−1^ *	300–3000 kJ kg^−1^ [4]
Temperature difference needed for storage, Δ*T*		25–1200 °C [4]	0–50 °C *	100–500 °C [4]
Volume change		~1% [4]	10–40% [5]	>1000% (at 1 atm) [4]
Complexity		Very simple	Simple	Complex
Maturity		Industrial scale	Pilot scale	Laboratory scale

* This work.

**Table 2:** Desired properties of porous silica–based phase change materials.

Type	Property	Value	Benefits
Thermal properties	Heat of fusion	High	Increased energy storage density
	Specific heat	High	
	Thermal conductivity	High	Increased power density, lower temperature gradients
	Melting point		Determines operating temperature
Physical properties	Volume change on transition	Low	Increases stability, minimizes leakage
	Vapor pressure	Low	Decreases evaporative loss of material
	Crystallization rate	High	Decreases the hysteresis between charging and discharging
	Supercooling degree	Low	
Chemical properties	Thermal & chemical stability	High	Increases life cycle
	Reactivity/corrosiveness	Low	
	Non-toxic, non-flammable, non-explosive	High	Increases safety and decreases system complexity
	Wettability & surface tension	High	Formation of shape stabilized materials with the silica matrix and higher PCM loading
Economic properties	Cost	Low	Improved economic efficiency and decreased risk
	Abundance	High	
	Environmental impact	Low	
